# Novel caries loci in children and adults implicated by genome-wide analysis of families

**DOI:** 10.1186/s12903-018-0559-6

**Published:** 2018-06-01

**Authors:** Manika Govil, Nandita Mukhopadhyay, Daniel E. Weeks, Eleanor Feingold, John R. Shaffer, Steven M. Levy, Alexandre R. Vieira, Rebecca L. Slayton, Daniel W. McNeil, Robert J. Weyant, Richard J. Crout, Mary L. Marazita

**Affiliations:** 1 Center for Craniofacial and Dental Genetics, Department of Oral Biology, School of Dental Medicine, University of Pittsburgh, Suite 500 Bridgeside Point, 100 Technology Drive, Pittsburgh, PA 15219 USA; 2 Department of Human Genetics, Graduate School of Public Health, University of Pittsburgh, Pittsburgh, PA USA; 3 Department of Biostatistics, Graduate School of Public Health, University of Pittsburgh, Pittsburgh, PA USA; 4 Department of Preventive and Community Dentistry, University of Iowa College of Dentistry, Iowa City, IA USA; 5 Department of Epidemiology, University of Iowa College of Public Health, Iowa City, IA USA; 6 Department of Pediatric Dentistry, School of Dentistry, University of Washington, Seattle, WA USA; 7 Dental Practice and Rural Health, West Virginia University School of Dentistry, Morgantown, WV USA; 8 Department of Psychology, Eberly College of Arts and Sciences, West Virginia University, Morgantown, WV USA; 9 Department of Dental Public Health and Information Management, School of Dental Medicine, University of Pittsburgh, Pittsburgh, PA USA; 10 Department of Periodontics, West Virginia University School of Dentistry, Morgantown, WV USA; 11 Clinical and Translational Science Institute, and Department of Psychiatry, School of Medicine, University of Pittsburgh, Pittsburgh, PA USA

**Keywords:** Dental genetics, Dental public health, Permanent dentition caries, Primary dentition caries, Non-parametric linkage, Genome-wide linkage study

## Abstract

**Background:**

Dental caries is a common chronic disease among children and adults alike, posing a substantial health burden. Caries is affected by multiple genetic and environmental factors, and prior studies have found that a substantial proportion of caries susceptibility is genetically inherited.

**Methods:**

To identify such genetic factors, we conducted a genome-wide linkage scan in 464 extended families with 2616 individuals from Iowa, Pennsylvania and West Virginia for three dental caries phenotypes: (1) **PRIM**: dichotomized as zero versus one or more affected primary teeth, (2) **QTOT1**: age-adjusted quantitative caries measure for both primary and permanent dentitions including pre-cavitated lesions, and (3) **QTOT2**: age-adjusted quantitative caries excluding pre-cavitated lesions. Genotyping was conducted for approximately 600,000 SNPs on an Illumina platform, pruned to 127,511 uncorrelated SNPs for the analyses reported here.

**Results:**

Multipoint non-parametric linkage analyses generated peak LOD scores exceeding 2.0 for eight genomic regions, but no LOD scores above 3.0 were observed. The maximum LOD score for each of the three traits was 2.90 at 1q25.3 for **PRIM**, 2.38 at 6q25.3 for **QTOT1**, and 2.76 at 5q23.3 for **QTOT2**. Some overlap in linkage regions was observed among the phenotypes. Genes with a potential role in dental caries in the eight chromosomal regions include *CACNA1E, LAMC2*, *ALMS1*, *STAMBP*, *GXYLT2, SLC12A2*, *MEGF10*, *TMEM181, ARID1B,* and, as well as genes in several immune gene families. Our results are also concordant with previous findings from association analyses on chromosomes 11 and 19.

**Conclusions:**

These multipoint linkage results provide evidence in favor of novel chromosomal regions, while also supporting earlier association findings for these data. Understanding the genetic etiology of dental caries will allow designing personalized treatment plans based on an individual’s genetic risk of disease.

**Electronic supplementary material:**

The online version of this article (10.1186/s12903-018-0559-6) contains supplementary material, which is available to authorized users.

## Background

Dental caries is one of the most common chronic diseases among children and adults alike. Childhood caries is associated with failure to thrive, and it can affect self-esteem and school performance [1]. For both children and adults, caries is associated with pain and loss of teeth, and caries may adversely impact growth and weight gain in children, as well as nutrition among adults, thereby negatively affecting quality of life.

Caries is known to have a genetic component. Detection of genetic factors is complicated by the fact that numerous diverse environmental factors influence the incidence and severity of this disease, including the microbiome, dietary habits, fluoride exposure, salivary factors and tooth structure.

Prior studies have shown that caries experience in humans is determined by genetic causes with heritability values between 20 and 60% [2–7]. To date, there have also been numerous studies investigating association of dental caries with candidate genes or with whole-genome Single Nucleotide Polymorphism (SNP) panels [8, 9]. The only previous genome-wide linkage study of caries was conducted using a panel of 392 microsatellite markers, on 46 extended Filipino families with 642 total individuals [10]. This study found suggestive linkage of low caries experience to chromosome regions 5q13.3, 14q11.2, and 13q27.1, and high caries experience to 13q31.1 and 14q24.3 However, results of previous studies have, in general, not been extensively replicated, possibly due to relatively small sample sizes [8, 9] and the enumeration of genetic factors is far from complete.

Our present study is the first to apply genome-wide multipoint linkage analysis to explore the genetic etiology of caries (whether in childhood or adulthood) using densely spaced SNPs on a population previously analyzed by genome-wide association. Genome-wide linkage analysis is a complementary strategy to genome-wide association analysis for gene-discovery. Whereas association identifies specific marker alleles correlated with the caries phenotype, linkage analysis strategies identify genomic regions shared between related individuals who show similar disease characteristics. The advantage of linkage analysis is that it makes full use of familial inheritance, is less sensitive to allelic heterogeneity, and, unlike association, can be used to detect rare disease-causing mutations. Furthermore, multipoint linkage utilizes genotypes from SNPs neighboring the test location, while association conducts tests at each location independently.

In this study, multipoint non-parametric linkage analysis was conducted, i.e., no assumptions were made with respect to the mode of inheritance of dental caries [11], and the analysis was, therefore, robust to uncertainty about the underlying genetic model. Empirical significance of the linkage signals was assessed across the genome by simulating multiple sets of genome-wide data such that the SNP genotypes were unlinked to caries status.

## Methods

### Study subjects and genotype data

The families and individuals included in this study are from western Pennsylvania, West Virginia, and Iowa. Subjects from Pennsylvania and West Virginia were ascertained through the Center for Oral Health Research in Appalachia (COHRA; [12]). Additional subjects from Pennsylvania were recruited under the University of Pittsburgh Dental Registry and DNA Repository (DRDR; [13]). Subjects from Iowa were recruited under two University of Iowa projects, the Iowa Fluoride Study (IFS; [14–17]) and Iowa Head Start (IHS; [18]). All subject recruitment and data collection was approved by site-specific Institutional Review Boards. Genotyping was conducted under the Gene Environment Association Studies Initiative (GENEVA) for approximately 600,000 SNPs on an Illumina platform (Human 610_Quadv1_B; Illumina, Inc., San Diego, CA, USA). All genotype and phenotype data is available on dbGaP (The database of Genotypes and Phenotypes; https://www.ncbi.nlm.nih.gov/gap; accession number phs000095.v3.p1). Details on genotyping and quality control protocols are also presented on dbGaP, or can be found in earlier studies [19, 20]. Table 1 summarizes the different subsets of data in terms of the sample available for this study. This study utilizes complete families, in other words, non-genotyped and non-phenotyped individuals also contribute to various aspects of the analysis. Prior studies primarily utilized unrelated individuals for conducting association analysis. The starting study sample comprised a total of 4727 self-reported non-Hispanic white individuals, of which 437 were unrelated, 1674 were in 558 two-parent and single offspring (trio) families, and 2616 were in 464 non-trio families. Approximately 76% of individuals were genotyped (Table 1).

**Table 1:** Starting sample size

Site	Current study starting sample			
	Unrelated	Trios^a^ (Individuals)	Non-trio pedigrees^b^ (Individuals)	Genotyped/Total
COHRA	29	162 (486)	452 (2549)	2209/3064
IFS	–	394 (1182)	4 (32)	964/1214
IHS	169	1 (3)	7 (29)	183/201
DRDR	239	1 (3)	1 (6)	235/248
Total	437	558 (1674)	464 (2616)	3591/4727

Note: ^a^Trios: Family structure of two parents and one child

^b^Non-trio pedigrees: Families with four or more members

### Definition of dental caries phenotypes

Caries scores were assessed on the COHRA, IFC, and IHS subjects in accordance with the COHRA study protocol [12]. For subjects in the DRDR study, we used caries scores abstracted from clinical records by dental students trained by Dr. Alexandre R. Vieira, who is a co-author on this manuscript.

We defined three dental caries phenotypes, one based on primary dentition (**PRIM**), and two that combine primary and permanent dentitions (**QTOT1**, **QTOT2**). **PRIM** was coded as a binary primary dentition caries phenotype based on the count of decayed and/or filled primary teeth (*dft***)** score. An individual with a *dft* score of 1 or more was designated as being affected. The primary teeth from all subjects with primary or mixed dentition were assessed for **PRIM**. These individuals included adults with over retained primary teeth. **QTOT1**, an age-adjusted quantitative caries phenotype, is based on the sum of the *dft* score (primary teeth) and *D*_*1*_*MFT* score (count of decayed, missing, and filled permanent teeth including white spot lesions). **QTOT1** scores were generated by adjusting this raw sum for age and age-squared effects using locally fitted splines. Scores for 113 individuals below 2 years of age and 5 individuals above 60 years, were excluded from linkage analysis due to a very low caries experience in the 0–2 years age group, and the presence of very few subjects above 60 years of age. Age-adjusted **QTOT2**, the second quantitative caries phenotype, is based on the sum of the *dft* score and the *D*_*2*_*MFT* score (count of decayed, missing, and filled permanent teeth excluding white spot lesions). Age-adjustment was performed as for the **QTOT1** phenotype; and **QTOT2** scores for 115 individuals between 0 and 2 years of age and 44 individuals above 60 years were set to missing.

### Data cleaning and preparation

Genetic map positions were generated for all SNPs. These genetic markers were filtered based on genotyping rates and Hardy-Weinberg proportions. The SNPs were then pruned for linkage disequilibrium (LD). SNPs with residual LD were clustered into super-markers. The procedures used for filtering SNPs, map creation, and LD-based SNP pruning and clustering are described below.

#### Genetic map creation

The Genetic Map Interpolator (GMI) program, [21] was used to calculate genetic map positions for all SNPs. Sex-averaged map positions were created for SNPs on chromosomes 1–22, and female map positions were created for SNPs on the X chromosome. In the GMI program, the physical basepair (bp) position of each SNP per March 2006 Build NCBI36/hg18 was transformed to the corresponding centiMorgan (cM) scale genetic map distance based on interpolation into the Rutgers Combined Linkage-Physical Map [22].

#### SNP filtering

In addition to the quality control and cleaning steps detailed on dbGap, we filtered SNPs on the basis of low genotyping success rate and deviation from Hardy-Weinberg equilibrium (HWE) proportions using the software PLINK [23]. SNPs with genotyping success rates below 95%, calculated using genotype data for all individuals, were excluded from analysis. Known genotypes of founders (i.e. those individuals in a family whose parents are not included in the study) and unrelated individuals were used to test SNPs for HWE proportions. The HWE proportions significance threshold was set at 10^− 5^ for rejecting the null hypothesis of no deviation from HWE proportions.

#### Linkage disequilibrium-based SNP pruning and clustering

The genotyping panel available to this study was designed for genome-wide association analysis. When conducting linkage analysis on densely spaced SNP marker loci, the presence of substantial marker-to-marker LD is known to inflate linkage signals, especially if parental genotypes are missing [24]. In this study, LD was removed in two stages. First, the set of quality-filtered SNPs were pruned using PLINK such that the LD r^2^ (a measure of LD based on the square of the correlation coefficient between loci) value among remaining SNPs fell below 20%. In PLINK, LD pruning consists of creating blocks of 50 consecutive SNPs followed by recursive removal of SNPs within blocks, until the LD r^2^ value among the remaining SNPs is below the desired threshold (20% in our case). Only the unrelated genotyped individuals in our data – pedigree founders and unrelated cases/controls -- were used to calculate LD in this step. Any remaining LD was then accounted for using LD-based clustering in Merlin [25]. In clustering, each block of consecutive SNPs that shows an r^2^ value greater than a specified threshold (in our case 10%), is analyzed collectively as a super-marker.

Table 2 summarizes the data processing steps undertaken to select SNPs for linkage analysis, and the samples that contributed information to specific parts of this data cleaning. After HWE filtering and LD-based pruning, 127,511 SNPs in low LD (pairwise r^2^ ≤ 20%) were retained. LD-based SNP clustering combined 92,495 SNPs into 20,634 super-markers, leaving 35,016 SNPs to be analyzed individually. The average genetic map distance between the final set of markers (super-marker index and singleton SNPs) is approximately 0.07 cM on the autosomes and 0.13 cM on the X-chromosome. Super-marker and singleton SNP allele frequencies were generated as maximum likelihood estimates using Merlin. The SNP clustering and allele frequency estimation steps utilized 1022 informative families.

**Table 2:** Sample for data cleaning

Procedure	Data
Low genotype rate filtering (PLINK)	3591 genotyped individuals
HWE testing (PLINK)	1839 genotyped (founders^a^ + unrelated)^b^
LD-based pruning (PLINK)	1839 genotyped (founders^a^ + unrelated)^b^
LD-based clustering and super-marker creation (Merlin)	1022 families (trios + non-trio pedigrees)^c^
Super-marker and SNP allele frequency estimation (Merlin)	1022 families (trios + non-trio pedigrees)^c^

Note: ^a^Founders: Individuals in a pedigree or trio whose parents are not included in the study. For example, both parents in a trio are founders. Also note that some of the larger multigenerational pedigrees may have more than two founders

^b^The counts of individuals differs from totals provided in Table 1 since not all founders and unrelated individuals were genotyped for this study

^c^Trios: Family structure of two parents and one child; non-trio pedigrees: families with four or more members

**Table 3:** Linkage analysis final sample

	PRIM	QTOT1	QTOT2
Total Non-trio Pedigrees	108	376	385
COHRA	106	372	373
IFS	1	4	4
IHS	1	–	7
DRDR	–	–	1
Total Individuals	687	2200	2243
Phenotyped	243 affected	1738	1756
Genotyped	483	1582	1604
Total informative relative pairs^a^	160	1026	1038
Median [Min, Max] pairs/pedigree	1 [1–6]	1 [1–24]	1 [1–24]
Sibling-pairs	100	599	609
Half-sibling pairs	39	228	228
Cousin pairs	21	73	73
Grandparent-grandchild	0	28	28
Avuncular	0	98	100

Note: ^a^PRIM: Affected relative pairs; QTOT1, QTOT2: phenotyped relative pairs

### Linkage analysis

Table 3 summarizes the sample of individuals used within the linkage analysis for the three traits. In the table are presented the number of informative pedigrees, individuals, and phenotyped relative pairs by relationship type that were included in NPL and QT linkage. A total of 160 relative pairs were informative for PRIM NPL. For QT linkage, the corresponding informative relative pair counts were 1026 and 1038 for QTOT1 and QTOT2.

#### Genome-wide linkage of PRIM

In non-parametric linkage (NPL) analysis, affected individuals within each pedigree are examined to detect whether affected relatives share genomic regions identical-by-descent (IBD) more often than expected due to their relatedness alone. This IBD sharing is tested at locations along each chromosome. Genome-wide NPL analysis was carried out for the **PRIM** phenotype using the S_All_ statistic [26] as implemented in Merlin [25].

#### Genome-wide linkage of QTOT1 and QTOT2

The quantitative trait (QT) regression-based linkage method, Merlin-regress, [27] was utilized to carry out analyses of the two quantitative phenotypes across autosomes. The QT linkage method is based on regressing estimated IBD sharing between relative pairs on the squared sums and differences of their phenotypes. It does not handle X-linked SNPs, hence the X chromosome was not analyzed for the two quantitative traits. Merlin-regress analyses required specification of a heritability parameter (set at 50% based on published estimates for *DMFT*) and sample-based means and variances for **QTOT1** and **QTOT2.** All results, NPL and QT linkage, are reported as LOD (logarithm of the odds of linkage) scores.

#### Empirical significance of observed linkage signals

The most commonly used LOD score threshold of 3.0 used to test for significant linkage (Morton) was derived for parametric linkage analysis of a single locus on a binary trait phenotype. Subsequent research (e.g. those reviewed in [28]) that address newer linkage methods such as whole-genome analysis, affected-relative pairs and multipoint calculations are also based on assumptions on the study data, that are rarely true in real-life. Therefore, to correct for multiple testing, we carried out a simulation study to assess the genome-wide significance thresholds for the NPL and QT regression LOD scores. In general, for a null simulation, hundreds of simulated genetic data sets are generated and analyzed to produce an empirical distribution of LOD scores. Since this process would be prohibitively time consuming given the study data, we used an adaptive approach to generate null distributions. The replicate pool method, Pseudo [29] was used to derive the empirical null distribution of NPL scores for PRIM. An initial pool of 100 simulated genotype data sets was generated for this study using Merlin (simulate option) followed by the pseudo-simulation of 100,000 NPL genome-scans to create the empirical distribution of unlinked NPL LOD scores. Pseudo was not utilized for the quantitative data simulations since QT-regression does not produce pedigree-specific LOD scores. For QTOT1 and QTOT2, 5000 data sets each were simulated and analyzed using Merlin.

#### Selection of linkage peak regions and etiologic genes

For super-markers, the NPL and QT LOD scores correspond to the index (first) SNP of each cluster. In the linkage scan for each phenotype, maximizing markers in regions with LOD score ≥ 2.0 were identified as linkage peaks. A support interval of one LOD drop was used for exploring genes under selected linkage peaks. The one LOD drop support interval is the interval where the LOD score is within one unit of its maximum.

Regions with LOD scores ≥1.0 were identified for trait. Overlap of linkage signal among the three traits was determined based on overlap of peak support intervals or secondary peaks(s) of at least 1.0 LODs, lying within the primary peak support interval. In the event peaks for multiple phenotypes overlapped, the resulting support intervals were reduced to the region of overlap.

Genes within these support intervals were examined for a potential etiologic role in dental caries incidence. Genes identified as causal would include, for example, genes related to blood glucose levels, secretory function of the salivary glands, and host immune response. Proximity of genes to SNPs corresponding to linkage peaks was determined by physical map positions obtained from UCSC Genome Browser corresponding to the March 2006 (NCBI36/hg18) Assembly [30]. When no genes were identified as potentially contributing to caries risk, we instead listed the gene closest to the SNP with the maximum observed LOD.

#### Comparison with prior published findings

A systematic search of literature was conducted to compile caries risk-conferring genes and genomic regions from previous studies utilizing some portion of our data, as well as from studies of other populations. Physical positions for these genes and genomic regions were then mapped to our linkage scans. Linkage regions with a LOD score of 1.0 or greater have been reported as indicative of concordance or replication, as appropriate.

### Sensitivity analysis

#### Effect of variation in parameter values on NPL statistics

For multifactorial diseases such as caries, the true underlying genetic model for disease is difficult to ascertain. In this study, model-free linkage methods were used to detect linkage. The QT methods are sensitive to the misspecification of the required programmatic input values. We conducted a sensitivity analysis for the heritability parameter (HP), since published literature provides a wide range of heritability values (40–60%), and our work utilized HP = 50%. In the sensitivity analysis, HP values were set at 40, and 60% for **QTOT1** and **QTOT2**.

Mega2 [31] was used to re-format and create input files for all the software used in data cleaning, LD-based pruning and clustering, genetic map creation, linkage analysis and data simulation.

## Results

### Study sample characteristics

**Fig. 1:**
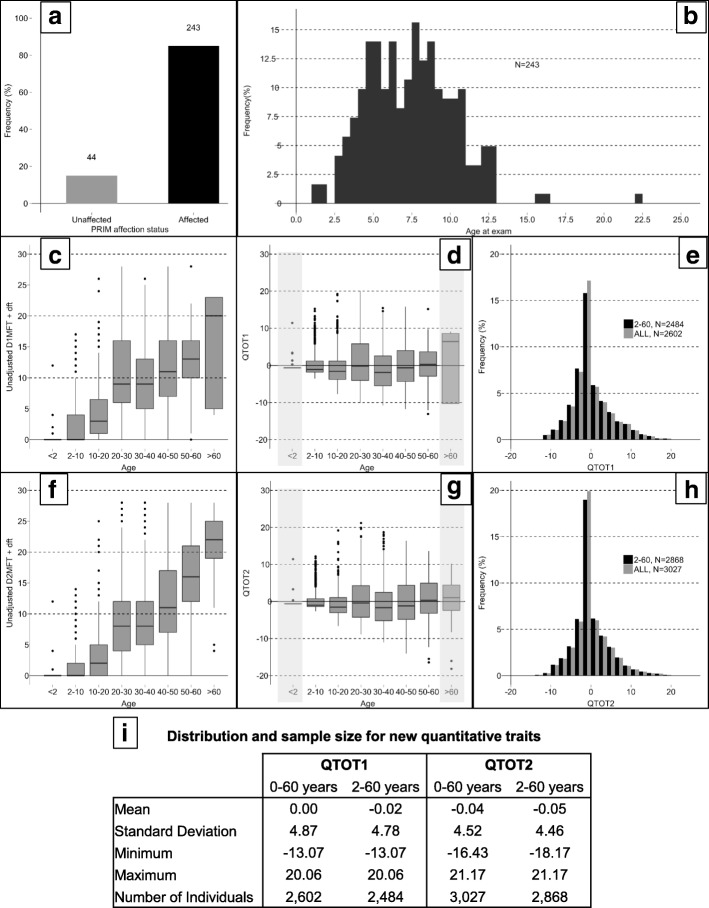
Distribution of (**a**) **PRIM** by binary affection status, (**b**) age at exam of individuals categorized as **PRIM** affected, (**c**) raw *dft* + D_1_MFT (**d**) age-adjusted **QTOT1,** (**e**) age-adjusted **QTOT1** for the full sample compared to the distribution for the 2–60 age group, (**f**) raw *dft* + D_2_MFT, (**g**) age-adjusted **QTOT2**, (**h**) age-adjusted QTOT2 for the full sample compared to the distribution for the 2–60 age group, and (**i**) mean, standard deviation, range and sample size for QTOT1 and QTOT2; shaded areas in panels D and G indicate individuals below the age of 2 and above 60 years with phenotypes excluded from quantitative trait linkage analysis

Figure 1 provides detailed information on the distribution of the three phenotypes, **PRIM** (panels A and B), **QTOT1** (panels C, D, E and I), and **QTOT2** (panels F, G, H and I). There were 287 individuals with known **PRIM** phenotypes (panel A), of which 243 individuals were affected for PRIM. Of these 243 subjects, 242 were 18 years or younger in age (panel B). Subjects with primary or mixed dentition included in the **PRIM** NPL analysis ranged from 15 months to 22.5 years of age, with a mean of 7.4 years. These subjects with primary dentition caries constitute mainly the youngest generation. The distribution of the raw caries index by decade, age-adjusted index by decade, and age-adjusted caries index within all phenotyped individuals compared to those between 2 and 60 years of age are shown for **QTOT1** (panels C, D, and E) and **QTOT2** (panels F, G, and H). The number of phenotyped individuals, range, mean and standard deviation are presented in panel I for both quantitative traits. A larger number of individuals were phenotyped for *D*_*2*_*MFT* as compared to *D*_*1*_*MFT* in this study. For **QTOT1** and **QTOT2**, there were 2484, and 2868 phenotyped individuals in the 2–60 age range. Both of the age-adjusted phenotypes follow an approximately normal distribution, with a mean of zero. The **QTOT1** and **QTOT2** mean and standard deviations for the 2–60 age group were included as distribution parameters within quantitative trait linkage.

### Linkage analysis

**Fig. 2:**
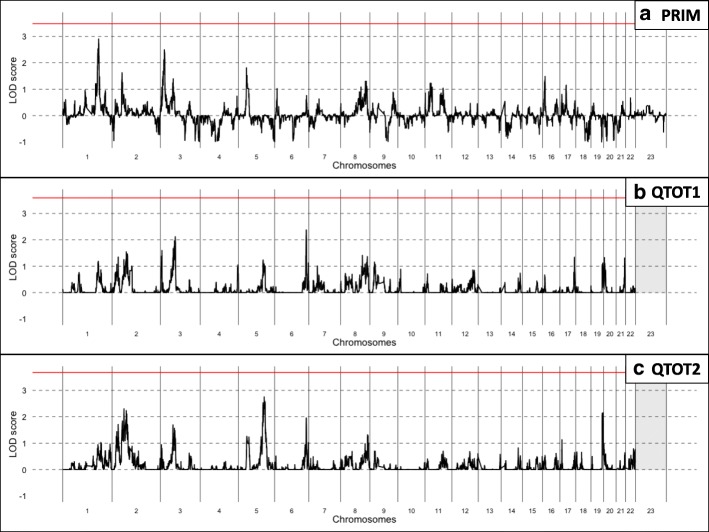
Genome-wide LOD scores: (**a**) **PRIM**, (**b**) **QTOT1**, (**c**) **QTOT2**. **PRIM** results include the X chromosome. The empirical genome-wide 0.05 significance levels are indicated in each panel with a solid (red, online) horizontal line

Figure 2 shows genome-wide LOD scores by SNP (or super-marker index SNP) for **PRIM**, **QTOT1**, and **QTOT2**. The empirical 5% genome-wide significance level, indicated as a solid horizontal line, was 3.48, 3.61, and 3.76 for **PRIM**, **QTOT1**, and **QTOT2**, respectively. Overlapping LOD score peaks for multiple phenotypes were observed in a few regions.

#### Highest LOD Score Regions

Table 4 presents peak LOD scores and 1-LOD support intervals. The SNP (or index SNP) with the maximum LOD value in each peak is identified along with its genomic location. Regions with maximum LOD ≥ 2.0 are shown ordered by chromosome, along with secondary peak(s) of at least 1.0 LODs, if observed for other traits. The highest LOD scores by trait were 2.90 for **PRIM**, 2.38 for **QTOT1**, and 2.76 for **QTOT2**. Detailed results for all SNPs that lie within the support region for peaks reported in Table 4 with a LOD score of 2.0 or more are provided in supplementary material [see Additional File 1].

**Table 4:** Linkage peaks in highest LOD score regions

Chr	Trait	Peak^a^			Support Interval (Mb)^b^ for Peak with LOD ≥ 2		Closest Genes within Support Interval^c^
		SNP	bp	LOD	Left	Right	
1	**QTOT1**	rs12096999	178,046,412	1.19			*CACNA1E*; *LAMC2*
1	**PRIM**	rs1281317	180,232,077	**2.90**	174.78	182.03	
2	**QTOT2**	rs7572396	59,893,993	**2.30**	58.63	64.29	*BCL11A* ^d^
2	**QTOT1**	rs13420242	71,117,276	1.55			*ALMS1*; *STAMBP*
2	**QTOT2**	rs831535	73,976,537	**2.10**	65.23	79.72	
3	**PRIM**	rs9842115	20,378,197	**2.50**	15.06	22.19	*KAT2B* ^d^
3	**QTOT1**	rs2044594	74,474,447	**2.12**	67.65	76.08	*GXYLT2*
5	**QTOT1**	rs11748635	123,232,224	1.24			*SLC12A2*; *MEGF10*; *IL* gene family
5	**QTOT2**	rs6866597	128,905,516	**2.76**	122.43	133.84	
6	**QTOT1**	rs240642	158,117,314	**2.38**	156.81	159.48	*TMEM181*; *ARID1B*
6	**QTOT2**	rs9295289	158,387,494	1.96			
19	**QTOT1**	rs11084325	59,424,868	1.12			*NLRP2*; *NLRP7*; *NLRP*, *KIR*, and *LILR* gene families
19	**QTOT2**	rs1671133	60,198,861	**2.15**	59.42	61.47	

Note: ^a^Novel regions with LOD ≥ 2.00, and secondary peaks ≥1.0 observed for the other phenotypes; peak LOD ≥ 2.00 shown in bold

^b^Support interval for LOD ≥ 2.00; Mb: 10^6^ (or 1 million) bp

^c^Genes with a potential role in caries incidence. If no such gene is identified, then the closest gene to the peak reported

^d^Unknown role in caries incidence; closest gene to the linkage peak SNP

For each linkage peak, the table also reports the closest gene, if found, with a potential role in caries incidence. For two of these peaks, one on chromosome 2 (**QTOT2**; LOD 2.30) and the other on chromosome 3 (**PRIM**; LOD 2.50), no such etiologic genes were identified within the support intervals. In these intervals, genes *BCL11A* (60.538–60.634 Mb) and *KAT2B* (20.056–20.171 Mb) were found to be closest to the respective LOD score peak SNPs. The genes within linkage peak regions that may play an etiologic role in dental caries are described in the sections below.

##### Chromosome 1

The highest LOD 2.90 across all three traits was observed on chromosome 1 for PRIM (Table 4). Under this peak, the *CACNA1E* (179.719–180.037 Mb) gene has been shown to be involved in glucose-evoked insulin secretion in mice [32]. Poor glycemic control has potential implications for increased caries risk in humans. Mutations in the *LAMC2* (181.422–181.481 Mb) laminin gene are known to cause non-Herlitz form of junctional epidermolysis bullosa, which includes hypodontia and dental caries among its phenotypes [33].

##### Chromosome 2

The second **QTOT2** peak on chromosome 2 includes the *ALMS1* (73.466–73.691 Mb) gene. Mutations in this gene causes Alström syndrome, where gingivitis and discolored enamel are two clinical phenotypes [34]. Individuals with mutations in the *STAMBP* (73.910–73.944 Mb) gene have been reported to have cleft palate and facial dysmorphology [35].

##### Chromosome 3

The *GXYLT2* (73.020–73.107 Mb) gene is located within the chromosome 3 **QTOT1** peak. *GXYLT2* acts on epidermal growth factor, which is expressed in human submandibular and parotid glands, and important for the maintenance of oroesophageal and gastric tissue.

##### Chromosome 5

The highest genome-wide quantitative trait LOD was observed for **QTOT2**. This **QTOT2** peak contains the *SLC12A2* (127.447–127.553 Mb) gene, whose protein product helps the movement of chloride ions in saliva, thereby assisting in salivary function. Also within the support interval are genes from the *IL* family, which code for cytokines involved in blood production and immune system function. Defects in these genes result in autoimmune diseases and immune deficiency. A third gene, *MEGF10* (126.654–126.825 Mb) has been implicated in MARDD (Myopathy, areflexia, respiratory distress, and dysphagia), with cleft palate as an associated phenotype [36].

##### Chromosome 6

The *TMEM181* (158.877–158.976 Mb) gene under the **QTOT1** linkage peak codes for a putative G-coupled protein receptor which mediates reaction to cytolethal distending toxins secreted by many pathogenic bacteria. *ARID1B* (157.141–157.572 Mb) mutations result in mental retardation along with minor teeth anomalies [37].

##### Chromosome 19

This region harbors several genes from the *NLRP*, *KIR*, and *LILR* immune gene families that code for various receptors within immune cells. *NLRP2* (60.170–60.204 Mb), and *NLRP7* (60.127–60.151 Mb) were closest to the peak.

#### Comparison with previous relevant signals

Table 5 shows regions reported by previous studies, where our current LOD score is 1.0 or greater. Two regions were found to contain genes reported in prior studies.

**Table 5:** Linkage signals with LOD ≥ 1 concordant with published findings

Study, Gene, phenotype	Highest observed LOD Score			
	SNP	bp	LOD	Trait
Genome-wide association, *MPPED2*, dichotomized *d*_*1*_*ft*^a^ in US children aged 3–12 (Shaffer et al. 2011)	rs1447267	30,643,586	1.23	PRIM
Association with gene set enrichment, *NLRP12*, dichotomized *d*_*1*_*ft*^1^ in children aged 3–12 (Wang et al. [38])	rs11084325	59,424,868	1.12	QTOT1
	rs1671133	60,198,861	2.15	QTOT2

Note: ^a^Dichotomized *d*_*1*_*ft* as used in our study

##### Chromosome 11

A **PRIM** LOD of 1.23 was observed 8500 bp from the *MPPED2* (30.338–30.558 Mb) gene. A suggestive association of primary teeth caries was reported by a previous study on 1305 children aged 3–12, some of whom are also part of this analysis (Shaffer et al., 2011). The phenotype was defined similarly to our PRIM phenotype.

##### Chromosome 19

A gene-set enrichment analysis study [38] reported an association of primary teeth caries to *NLRP12* (58.989–59.019 Mb) in 1142 children aged 3–13, a subset of whom are also included in our study. **QTOT1** and **QTOT2** LOD scores ≥1.0 were observed 0.4–1.2 Mb from this gene.

### Sensitivity analysis

**Fig. 3:**
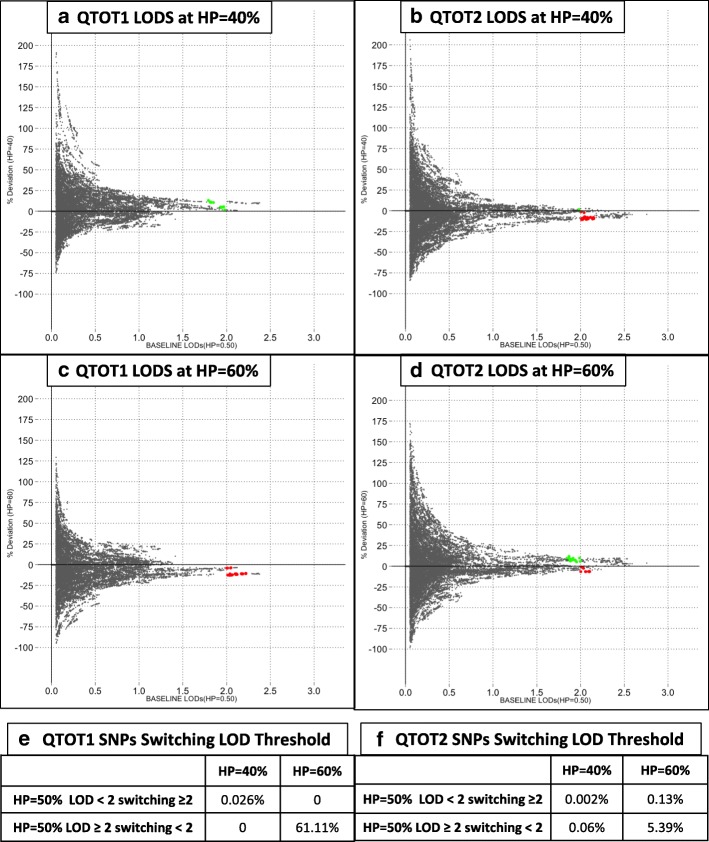
Sensitivity of QT LOD score to changes in HP. Panels (**a**) and (**c**) are for **QTOT1**, and (**b**) and (**d**) are for **QTOT2**. In each scatterplot, the x-axis represents LOD scores reported in this paper, using HP = 50%. In panels (**a**) and (**b**), the y-axis represents LOD scores for HP = 40%. In panels (**c**) and (**d**), y-axis represents LOD scores calculated with HP = 60%. Panels E and F show the proportion (%) of SNPs switching from LOD ≤ 2.0 to LOD ≥ 2.0 for **QTOT1** and **QTOT2**

For each of the two quantitative traits, **QTOT1** and **QTOT2**, Fig. 3, panels A, B, C, and D show the percentage deviation of LODs obtained using HPs of 40% or 60% from baseline LODs produced with an HP of 50%. These deviations are plotted on the y-axis against the corresponding baseline LOD (x-axis). The red points indicate SNPs for which baseline LODs of 2.0 or greater dropped below 2.0 when the HP value was changed. Conversely, the green points show SNP positions with baseline LODs below 2.0, which subsequently switched to a score of 2.0 or more with a change in HP. Percentage deviations where the baseline LODs were between 0 and 0.05 are not presented in panels A through D. Within this range, the change in LOD combined across HP = 40% and HP = 60% ranges from − 0.04 to 0.11 LOD for **QTOT1**. For **QTOT2**, the corresponding range is − 0.05 to 0.1 LOD. Although in percentage terms they represent exponential changes as compared to the baseline, none of the deviations in the 0 to 0.5 baseline LOD score range result in the LOD score approaching significance. Panels E and F break down for each trait, the percentage of all SNPs that drop below—or exceed—the 2.0 LOD score threshold with a change in HP. For both traits, a decrease in HP to 40% results in a minimal percentage of SNPs changing status (be it an increase or decrease in LOD score). In contrast, SNPs with LOD scores of 2.0 or greater at HP 50% are more likely to drop below the 2.0 LOD threshold when the HP is increased to 60%.

Table 6 presents the change in **QTOT1** and **QTOT2** LOD scores due to a change in HP for only the linkage peaks reported in Table 4. All LOD score peaks, except for one, remain above 2.0 despite changes in HP.

**Table 6:** Comparison of reported peaks in HP sensitivity analysis

Trait	Chr	SNP	A HP = 50%^a^	B HP = 40% [B-A]	C HP = 60% [C-A]
QTOT1	3	rs2044594	2.12	2.14 [0.02]	2.04 [−0.08]
	6	rs240642	2.38	2.62 [0.24]	2.10 [−0.28]
QTOT2	2	rs7572396	2.30	2.20 [−0.10]	2.36 [0.06]
	2	rs831535	2.10	2.16 [0.06]	2.00 [−0.10]
	5	rs6866597	2.76	2.64 [−0.12]	2.86 [0.10]
	19	rs1671133	2.15	1.95 [−0.20]	2.28 [0.13]

Note: ^a^These values are the peak LOD score results reported in Table 4 for **QTOT1** and **QTOT2**

## Discussion

To our knowledge, this study was the first to apply genome-wide multipoint linkage analysis to explore the genetic etiology of caries using densely spaced SNPs.

We defined two new quantitative phenotypes which combine childhood and adulthood caries indices while also accounting for variability by age. The linkage findings in this study nominated genes on six chromosomes (1, 2, 3, 5, 6, and 19) with potential involvement in caries etiology. Some of the genes are known to cause syndromes with a dental or oral phenotype, while others have a role to play in human immune and host defense response, blood glucose levels, and secretory function of the salivary glands all of which may have a potential impact on incidence of dental caries (see, for example, Carneiro et al. (2015) for the relationship between diabetes and dental caries). After a comprehensive review of the literature, we also detected linkage to regions on chromosomes 11 and 19 previously reported as associated to caries.

As expected, we do not recapitulate all findings from all prior association studies published by our group although this linkage study and the previous association studies utilized data from the same sources (i.e., COHRA, IHS, DRDR, IFS). As mentioned previously, linkage and association are complementary strategies for gene-discovery. In linkage, similarities and differences in pairs of phenotypes are modeled in terms of genetic similarity over related pairs from families. In association, this modeling is performed at the level of individuals. Our linkage uses multi-point analysis, i.e., the LOD score at any specific location is influenced by linkage at neighboring loci. Association generally uses a set of independent one-locus tests. Finally, as described in methods, this study differs from prior published work, both in the number, and the type (in terms of family composition) of individuals included in the analysis. Linkage utilizes family data and all related pairs (affected or phenotyped) within a pedigree whereas association generally is conducted on unrelated cases and controls, or at most parent-offspring trios.

The genotyping panel was designed for association analyses, and therefore, is far denser than a linkage SNP panel. Although dense bi-allelic SNP panels may allow extraction of more information, a concern for this study was existing linkage disequilibrium between SNPs. We pruned SNPs based on marker-to-marker LD, and then exploited any remaining LD among the pruned set to create clusters which served as polymorphic markers. Despite the pruning and clustering, our analysis was conducted on a much denser set of markers (35,016 SNPs and 20,634 super-markers) compared to a typical linkage panel with 6000 SNPs. The use of multi-allelic super-markers also had the potential of increasing power of linkage studies in such a setting.

Genome-wide significance for each phenotype was empirically assessed through a series of simulations, which provides an approximation of the true underlying distribution of a statistic since not all features of the data can be completely replicated. In an exploratory study, adhering strictly to genome-wide significance thresholds may be overly conservative. Furthermore, of the 4727 subjects, only 2616 contributed to the linkage analysis, providing a comparatively small number of relative pairs given the large sample size.

The sensitivity analysis conducted for the parameter HP explores the impact of parameter value selection on a model-free QT method. The results from this analysis indicated that the non-parametric quantitative trait linkage method, as implemented in Merlin, was robust to variation in HP, and that changing the HP parameter had a minimal impact on LOD scores. Even more importantly, the linkage peaks were insensitive to parameter misspecification.

Environmental factors are not accounted for in this study due to unavailability of such data on many of our subjects, which would have drastically reduced the cohort size. We also did not attempt to analyze gene-by-gene interaction. The available methods for detection of gene-gene interaction that are applicable to our study design are computationally complex, thus making whole-genome interaction analysis beyond the scope of the current work (e.g. see the review of the various classes of interaction detection methods by Li [39]).

## Conclusions

This study presents two new quantitative measures for dental caries which combine both the primary and permanent dentition, while adjusting for age effects. Genes identified in peak linkage regions underline the importance of exploring potential relationships between caries and other traits. We did not include environmental factors in this study. The interaction between putative caries risk conferring genes and factors including fluoride exposure, dietary habits, and the microbiome need to be investigated, as do interactions between the genes themselves. From a clinical perspective, individuals would be at an elevated lifetime risk of developing caries in both primary and permanent dentition, given increased genetic susceptibility. Understanding the genetic etiology of dental caries will allow health providers to design personalized treatment plans based on an individual’s genetic risk of disease.

## Additional file

Additional file 1: SNPs within support regions of reported peaks**.** Detailed results for all SNPs that lie within the support region for peaks with a LOD score of 2.0 or more, as summarized in Table 4 of the main paper. (DOCX 97 kb)

## Abbreviations

bp: basepair
cM: centiMorgan
COHRA: Center for Oral Health Research in Appalachia
D_1_MFT: Count of decayed, missing, and filled permanent teeth including white spot lesions
D_2_MFT: Count of decayed, missing, and filled permanent teeth excluding white spot lesions
dbGaP: The database of Genotypes and Phenotypes
dft: Count of decayed and/or filled primary teeth (dft) score
DRDR: University of Pittsburgh Dental Registry and DNA Repository
GENEVA: Gene Environment Association Studies Initiative
GMI: Genetic Map Interpolator
HP: Heritability Parameter
HWE: Hardy-Weinberg Equilibrium
IBD: Identical-By-Descent
IFS: Iowa Fluoride Study
IHS: Iowa Head Start
LD: Linkage Disequilibrium
LOD: Logarithm of the odds of linkage
MARDD: Myopathy, areflexia, respiratory distress, and dysphagia
Mb: 10^6^ (or 1 million) bp
NPL: Non-Parametric Linkage
PRIM: New binary dental caries phenotype based on dft
QT: Quantitative Trait
QTOT1: New age-adjusted quantitative caries phenotype based on the sum of the dft score and D_1_MFT score
QTOT2: New age-adjusted quantitative caries phenotype based on the sum of the dft score and D_2_MFT score
r^2^: A measure of LD based on the square of the correlation coefficient between loci)
SNP: Single Nucleotide Polymorphism
