# Supplementary material for: Novel caries loci in children and adults implicated by genome-wide analysis of families
Source: BMC Oral Health. 2018 Jun 1;18:98. doi: 10.1186/s12903-018-0559-6 (PMC5984765; doi:10.1186/s12903-018-0559-6)
Supplement: Supplementary file 1 — SNPs within support regions of reported peaks. Detailed results for all SNPs that lie within the support region for peaks with a LOD score of 2.0 or more, as summarized in Table 4 of the main paper. (DOCX 97 kb) [file 12903_2018_559_MOESM1_ESM.docx]

**Supplement 1: SNPs within support regions of reported peaks**

Detailed results are provided here for all SNPs that lie within the support region for peaks with a LOD score of 2.0 or more, as summarized in Table 4 of the main paper. Peaks ≥ 2.0 as reported in Table 4, are in boldface in the tables below.

**Chromosome 1, PRIM, peak LOD 2.90, support interval 174.78 Mb - 182.03 Mb**

| **SNP** | | **BP** | | **LOD** | | **SNP** | **BP** | **LOD** |
| --- | --- | --- | --- | --- | --- | --- | --- | --- |
| rs2206510 | | 174780160 | | 1.941 | | rs10753218 | 178823474 | 2.192 |
| rs2294649 | | 174786394 | | 1.958 | | rs12079690 | 178835343 | 2.194 |
| rs10798473 | | 174913006 | | 2.082 | | rs7556184 | 178924588 | 2.214 |
| rs7523235 | | 175102195 | | 2.36 | | rs16856420 | 179003051 | 2.221 |
| rs9662701 | | 175650659 | | 2.361 | | rs12035742 | 179031991 | 2.226 |
| rs10798522 | | 175709680 | | 2.361 | | rs12047845 | 179036943 | 2.226 |
| rs1252068 | | 175868691 | | 2.368 | | rs2271668 | 179115704 | 2.238 |
| rs16851869 | | 176017129 | | 2.425 | | rs12118635 | 179142353 | 2.243 |
| rs9970167 | | 176025772 | | 2.439 | | rs7515553 | 179156796 | 2.241 |
| rs10798572 | | 176057733 | | 2.481 | | rs3737062 | 179198131 | 2.236 |
| rs10159204 | | 176182121 | | 2.518 | | rs3789357 | 179286940 | 2.281 |
| rs1984159 | | 176184788 | | 2.52 | | rs4652553 | 179293327 | 2.284 |
| rs10913472 | | 176196177 | | 2.526 | | rs4651084 | 179339941 | 2.268 |
| rs12747656 | | 176198131 | | 2.529 | | rs6425668 | 179386458 | 2.128 |
| rs6425457 | | 176204064 | | 2.53 | | rs3856080 | 179401497 | 2.066 |
| rs1336779 | | 176234820 | | 2.525 | | rs3904753 | 179405504 | 2.064 |
| rs12409266 | | 176237621 | | 2.524 | | rs7523662 | 179414537 | 2.06 |
| rs16852358 | | 176257878 | | 2.521 | | rs3827707 | 179426082 | 2.053 |
| rs2185391 | | 176272848 | | 2.516 | | rs7550430 | 179427776 | 1.984 |
| rs12081217 | | 176306293 | | 2.479 | | rs11580052 | 179784231 | 2.329 |
| rs12354181 | | 176686742 | | 2.439 | | rs199937 | 179861088 | 2.596 |
| rs387933 | | 176691755 | | 2.393 | | rs175338 | 179871788 | 2.639 |
| rs4430291 | | 177143205 | | 2.289 | | rs199979 | 179891388 | 2.645 |
| rs7539958 | | 177179894 | | 2.272 | | rs6680280 | 179896241 | 2.646 |
| rs7522133 | | 177194050 | | 2.109 | | rs2280868 | 180008434 | 2.68 |
| rs10913662 | | 177210199 | | 2.048 | | rs704326 | 180026280 | 2.728 |
| rs12028381 | | 177216941 | | 2.002 | | rs602009 | 180141290 | 2.827 |
| rs6697180 | | 177233852 | | 2.017 | | rs12135968 | 180170131 | 2.87 |
| rs12066589 | | 177277569 | | 2.044 | | rs2262513 | 180180910 | 2.884 |
| rs12022201 | | 177305667 | | 2.061 | | rs1281292 | 180221885 | 2.894 |
| rs16853647 | | 177329120 | | 2.07 | | **rs1281317** | **180232077** | **2.905** |
| rs2171958 | | 177443021 | | 2.135 | | rs2236536 | 180325284 | 2.829 |
| rs4651024 | | 177578725 | | 2.265 | | rs3845452 | 180370812 | 2.731 |
| rs4651044 | | 177886288 | | 2.313 | | rs10911031 | 180440334 | 2.731 |
| rs2504057 | | 177953882 | | 2.337 | | rs1779821 | 180443279 | 2.731 |
| rs11576953 | | 177996669 | | 2.345 | | rs7546783 | 180474434 | 2.731 |
| rs10913887 | | 178017270 | | 2.354 | | rs10911049 | 180528618 | 2.731 |
| rs12096999 | | 178046412 | | 2.296 | | rs6703548 | 180558058 | 2.731 |
| rs7536561 | | 178510147 | | 2.16 | | rs10797768 | 180579099 | 2.731 |
| **SNP** | **BP** | | **LOD** | |  |  |  |  |
| rs497915 | 180673277 | | 2.731 | |  |  |  |  |
| rs12744324 | 180689290 | | 2.731 | |  |  |  |  |
| rs155552 | 180691608 | | 2.731 | |  |  |  |  |
| rs490531 | 180797401 | | 2.73 | |  |  |  |  |
| rs12757998 | 180805101 | | 2.729 | |  |  |  |  |
| rs533259 | 180815642 | | 2.729 | |  |  |  |  |
| rs1148769 | 180872570 | | 2.667 | |  |  |  |  |
| rs4652741 | 180893361 | | 2.61 | |  |  |  |  |
| rs12737684 | 180941601 | | 2.436 | |  |  |  |  |
| rs7554927 | 181033121 | | 2.27 | |  |  |  |  |
| rs4651133 | 181155420 | | 2.27 | |  |  |  |  |
| rs6676271 | 181196009 | | 2.27 | |  |  |  |  |
| rs12082934 | 181197955 | | 2.27 | |  |  |  |  |
| rs4652790 | 181508518 | | 2.269 | |  |  |  |  |
| rs12734496 | 181555214 | | 2.268 | |  |  |  |  |
| rs3842897 | 181770287 | | 2.264 | |  |  |  |  |
| rs3845461 | 181798823 | | 2.254 | |  |  |  |  |
| rs3845466 | 181818383 | | 2.247 | |  |  |  |  |
| rs10911412 | 181929052 | | 2.198 | |  |  |  |  |
| rs10494566 | 181957090 | | 2.176 | |  |  |  |  |
| rs2500097 | 182031682 | | 1.957 | |  |  |  |  |

**Chromosome 2, QTOT2, peak LOD 2.30, support interval 58.63 Mb - 64.29 Mb**

| **SNP** | **BP** | **LOD** | **SNP** | **BP** | **LOD** |
| --- | --- | --- | --- | --- | --- |
| rs12618801 | 58632908 | 1.421 | rs2419643 | 60171562 | 2.048 |
| rs17049587 | 58684715 | 1.452 | rs4672388 | 60452176 | 1.774 |
| rs17269126 | 58685911 | 1.453 | rs243035 | 60454880 | 1.756 |
| rs10865306 | 58766643 | 1.58 | rs733628 | 60556270 | 1.789 |
| rs12986742 | 58828647 | 1.576 | rs6729815 | 60577176 | 1.789 |
| rs4140883 | 58933042 | 1.526 | rs10184550 | 60582798 | 1.782 |
| rs6758100 | 58968243 | 1.524 | rs356982 | 60652625 | 1.764 |
| rs2192453 | 59021061 | 1.846 | rs842767 | 60722118 | 1.727 |
| rs2863301 | 59053351 | 1.859 | rs10198826 | 60763537 | 1.739 |
| rs2192463 | 59058410 | 1.864 | rs7589998 | 60777524 | 1.741 |
| rs6704661 | 59082006 | 1.884 | rs17537750 | 60786511 | 1.742 |
| rs11687358 | 59092872 | 1.892 | rs7576218 | 60821465 | 1.756 |
| rs9309307 | 59113811 | 1.949 | rs11678166 | 61011224 | 1.571 |
| rs7590342 | 59113908 | 1.949 | rs17541618 | 61408192 | 1.52 |
| rs10176695 | 59125286 | 1.974 | rs4387777 | 61483139 | 1.535 |
| rs991964 | 59136095 | 2.023 | rs2421333 | 61705751 | 1.679 |
| rs1541546 | 59247443 | 2.067 | rs7574498 | 62084474 | 1.64 |
| rs2192567 | 59310127 | 2.141 | rs6719628 | 62265706 | 1.654 |
| rs17644631 | 59434240 | 2.093 | rs4672482 | 62301903 | 1.66 |
| rs4432437 | 59459080 | 2.08 | rs4563212 | 62356521 | 1.607 |
| rs7577424 | 59464215 | 2.078 | rs6713620 | 62465549 | 1.626 |
| rs12464531 | 59527034 | 2.006 | rs13412212 | 62516208 | 1.668 |
| rs2160142 | 59553433 | 1.992 | rs4350731 | 62553294 | 1.642 |
| rs6709829 | 59590284 | 1.992 | rs10186788 | 62566799 | 1.624 |
| rs721250 | 59630520 | 1.991 | rs4233979 | 62581406 | 1.63 |
| rs17647256 | 59644444 | 1.993 | rs4608500 | 62617751 | 1.629 |
| rs11125800 | 59646468 | 1.993 | rs360791 | 62770760 | 1.486 |
| rs13429851 | 59670995 | 2.04 | rs13431584 | 63368155 | 1.437 |
| rs17595541 | 59714432 | 2.179 | rs17028375 | 64165646 | 1.401 |
| **rs7572396** | **59893993** | **2.305** | rs10496107 | 64222840 | 1.392 |
| rs2419405 | 60004686 | 2.044 | rs6710681 | 64266725 | 1.39 |
| rs10175755 | 60149146 | 2.047 | rs2217968 | 64274753 | 1.386 |
| rs17007458 | 60155323 | 2.047 | rs11125993 | 64292394 | 1.367 |
| rs13394010 | 60156250 | 2.047 |  |  |  |

**Chromosome 2, QTOT2, peak LOD 2.10, support interval 65.23 Mb – 79.72 Mb**

| **SNP** | | **BP** | | **LOD** | | **SNP** | | **BP** | | **LOD** | |
| --- | --- | --- | --- | --- | --- | --- | --- | --- | --- | --- | --- |
| rs11890484 | | 65232537 | | 1.317 | | rs17033180 | | 67266758 | | 1.641 | |
| rs268134 | | 65461867 | | 1.244 | | rs2062203 | | 67349940 | | 1.596 | |
| rs10451656 | | 65568948 | | 1.287 | | rs13394751 | | 67410529 | | 1.592 | |
| rs840947 | | 65570120 | | 1.285 | | rs17033537 | | 67485901 | | 1.59 | |
| rs840952 | | 65573445 | | 1.298 | | rs658602 | | 67540293 | | 1.588 | |
| rs6744773 | | 65610006 | | 1.299 | | rs1521043 | | 67569534 | | 1.592 | |
| rs3911862 | | 65613175 | | 1.3 | | rs17033681 | | 67577069 | | 1.592 | |
| rs1673459 | | 65664092 | | 1.301 | | rs17033691 | | 67579263 | | 1.607 | |
| rs6546167 | | 65692358 | | 1.303 | | rs4671197 | | 67806315 | | 1.503 | |
| rs11682811 | | 65735129 | | 1.304 | | rs1346870 | | 67842579 | | 1.617 | |
| rs9678488 | | 65777461 | | 1.196 | | rs1346869 | | 67842801 | | 1.617 | |
| rs17030670 | | 65788661 | | 1.205 | | rs730424 | | 67845579 | | 1.621 | |
| rs6546174 | | 65788897 | | 1.205 | | rs5004016 | | 67848031 | | 1.625 | |
| rs13407769 | | 65802663 | | 1.251 | | rs1897177 | | 67870180 | | 1.649 | |
| rs10167906 | | 66142167 | | 1.235 | | rs7602687 | | 67883207 | | 1.545 | |
| rs11684249 | | 66153166 | | 1.194 | | rs6715551 | | 67889466 | | 1.616 | |
| rs4671723 | | 66388259 | | 1.233 | | rs7592215 | | 67904959 | | 1.735 | |
| rs2946370 | | 66445915 | | 1.255 | | rs1032807 | | 68019584 | | 1.631 | |
| rs10203407 | | 66552300 | | 1.337 | | rs905487 | | 68041496 | | 1.634 | |
| rs6705285 | | 66562972 | | 1.352 | | rs17034835 | | 68089300 | | 1.675 | |
| rs6722685 | | 66577646 | | 1.391 | | rs7558466 | | 68103800 | | 1.699 | |
| rs2192954 | | 66631025 | | 1.555 | | rs7582426 | | 68120695 | | 1.803 | |
| rs10184250 | | 66657397 | | 1.577 | | rs921248 | | 68189174 | | 1.777 | |
| rs10490188 | | 66667741 | | 1.589 | | rs17034987 | | 68192117 | | 1.775 | |
| rs4671159 | | 66750877 | | 1.565 | | rs4078978 | | 68213401 | | 1.742 | |
| rs2192955 | | 66783965 | | 1.544 | | rs3732042 | | 68373381 | | 1.731 | |
| rs11126093 | | 66797551 | | 1.532 | | rs10179521 | | 68396798 | | 1.728 | |
| rs12622301 | | 66804355 | | 1.528 | | rs6717329 | | 68409204 | | 1.727 | |
| rs6748591 | | 66807867 | | 1.525 | | rs7602170 | | 68420563 | | 1.726 | |
| rs2081397 | | 66813423 | | 1.493 | | rs7595037 | | 68500599 | | 1.748 | |
| rs11126099 | | 66898646 | | 1.476 | | rs4854455 | | 68519372 | | 1.695 | |
| rs6546266 | | 66900369 | | 1.476 | | rs3969864 | | 68755112 | | 1.714 | |
| rs1559600 | | 66914658 | | 1.466 | | rs13000978 | | 68787405 | | 1.719 | |
| rs11126103 | | 67033691 | | 1.462 | | rs2292903 | | 68868024 | | 1.713 | |
| rs1548900 | | 67058133 | | 1.46 | | rs7604154 | | 68980468 | | 1.714 | |
| rs6727258 | | 67093532 | | 1.455 | | rs11894848 | | 68986244 | | 1.705 | |
| rs12993985 | | 67119483 | | 1.703 | | rs2312075 | | 68991760 | | 1.692 | |
| rs12622277 | | 67122799 | | 1.699 | | rs10191936 | | 68992530 | | 1.69 | |
| rs12996858 | | 67263616 | | 1.667 | | rs11126202 | | 68994331 | | 1.879 | |
| **SNP** | **BP** | | **LOD** | | **SNP** | | **BP** | | **LOD** | |  |
| rs4462818 | 69061806 | | 1.857 | | rs17616999 | | 71675355 | | 1.926 | |  |
| rs4435491 | 69182387 | | 1.826 | | rs2303606 | | 71692105 | | 1.935 | |  |
| rs4854547 | 69196037 | | 1.822 | | rs11897583 | | 71701920 | | 1.935 | |  |
| rs4358156 | 69208204 | | 1.819 | | rs13387541 | | 71718795 | | 1.93 | |  |
| rs7599536 | 69250457 | | 1.831 | | rs227782 | | 71720350 | | 1.924 | |  |
| rs4581932 | 69287184 | | 1.891 | | rs11897186 | | 71762871 | | 1.926 | |  |
| rs17570697 | 69608069 | | 1.861 | | rs1896496 | | 71791696 | | 1.931 | |  |
| rs17036760 | 69620677 | | 1.865 | | rs1427688 | | 71833962 | | 1.935 | |  |
| rs6759804 | 69665573 | | 1.895 | | rs10181010 | | 71835392 | | 1.935 | |  |
| rs13412112 | 69843794 | | 1.876 | | rs1459259 | | 71878478 | | 1.941 | |  |
| rs13391033 | 69922649 | | 1.679 | | rs12165184 | | 71940479 | | 1.971 | |  |
| rs11126264 | 70426197 | | 1.668 | | rs9973384 | | 71989836 | | 1.978 | |  |
| rs2169843 | 70469634 | | 1.659 | | rs12052842 | | 72020644 | | 1.983 | |  |
| rs1382454 | 70478701 | | 1.655 | | rs4852833 | | 72090437 | | 1.983 | |  |
| rs12996793 | 70503279 | | 1.634 | | rs2215801 | | 72176542 | | 1.95 | |  |
| rs1448927 | 70555741 | | 1.623 | | rs975612 | | 72242842 | | 1.938 | |  |
| rs432203 | 70618196 | | 1.59 | | rs11126387 | | 73021452 | | 1.943 | |  |
| rs12466990 | 70684136 | | 1.593 | | rs755409 | | 73024238 | | 1.942 | |  |
| rs1024578 | 70726943 | | 1.597 | | rs17504249 | | 73028356 | | 1.941 | |  |
| rs758062 | 70749570 | | 1.566 | | rs11898850 | | 73053127 | | 1.962 | |  |
| rs11684667 | 70878068 | | 1.75 | | rs7557285 | | 73296320 | | 1.923 | |  |
| rs6712273 | 70882947 | | 1.762 | | rs11893547 | | 73304912 | | 1.931 | |  |
| rs10178512 | 70890780 | | 1.788 | | rs1653259 | | 73953070 | | 2.092 | |  |
| rs741326 | 70912343 | | 1.915 | | rs862792 | | 73965922 | | 2.056 | |  |
| rs6748323 | 70955355 | | 1.922 | | rs7581248 | | 73973016 | | 2.099 | |  |
| rs10165187 | 71021085 | | 1.902 | | rs10176602 | | 73974293 | | 2.099 | |  |
| rs10469959 | 71055921 | | 1.856 | | rs13415017 | | 73975731 | | 2.098 | |  |
| rs2041689 | 71056850 | | 1.889 | | **rs831535** | | **73976537** | | **2.104** | |  |
| rs1861074 | 71083241 | | 1.961 | | rs13411881 | | 74005208 | | 2.06 | |  |
| rs10196871 | 71088542 | | 1.982 | | rs4852323 | | 74037181 | | 2.002 | |  |
| rs13420242 | 71117276 | | 2.193 | | rs7608165 | | 74055981 | | 1.794 | |  |
| rs1458868 | 71229709 | | 2.134 | | rs828867 | | 74187970 | | 1.719 | |  |
| rs12615297 | 71285393 | | 2.235 | | rs3099824 | | 74196075 | | 1.676 | |  |
| rs4852787 | 71560205 | | 2.165 | | rs828860 | | 74264789 | | 1.577 | |  |
| rs12998057 | 71562240 | | 2.11 | | rs828863 | | 74305767 | | 1.509 | |  |
| rs12469262 | 71587090 | | 2.206 | | rs2191309 | | 74370759 | | 1.351 | |  |
| rs2303596 | 71633723 | | 2.109 | | rs11678377 | | 74823901 | | 1.428 | |  |
| rs17007067 | 71645085 | | 2.034 | | rs2021753 | | 74824429 | | 1.405 | |  |
| rs10183967 | 71650684 | | 1.918 | | rs13405664 | | 74843257 | | 1.334 | |  |

| **SNP** | **BP** | **LOD** | **SNP** | **BP** | **LOD** |
| --- | --- | --- | --- | --- | --- |
| rs3771790 | 74924075 | 1.374 | rs17012947 | 76694958 | 1.31 |
| rs3771781 | 74938671 | 1.373 | rs1474177 | 76697534 | 1.314 |
| rs1869340 | 74955684 | 1.382 | rs13426498 | 76701021 | 1.319 |
| rs17010504 | 74969195 | 1.375 | rs12621435 | 76747364 | 1.381 |
| rs10496197 | 74974221 | 1.372 | rs17013020 | 76768478 | 1.407 |
| rs17010519 | 74995810 | 1.425 | rs6745127 | 76771646 | 1.475 |
| rs17010698 | 75134562 | 1.568 | rs13034829 | 76772575 | 1.484 |
| rs2422094 | 75136586 | 1.57 | rs13415879 | 76793083 | 1.716 |
| rs3771833 | 75220445 | 1.6 | rs4853265 | 76796318 | 1.638 |
| rs3771846 | 75243000 | 1.586 | rs12470091 | 76802033 | 1.484 |
| rs10168411 | 75294108 | 1.377 | rs10210863 | 76802609 | 1.481 |
| rs2422150 | 75322495 | 1.407 | rs10172954 | 76810878 | 1.447 |
| rs10490307 | 75335731 | 1.395 | rs12713881 | 76830134 | 1.358 |
| rs10490308 | 75335883 | 1.394 | rs13412373 | 76937749 | 1.399 |
| rs7565730 | 75407540 | 1.353 | rs10196177 | 76939260 | 1.432 |
| rs6749524 | 75441738 | 1.347 | rs7577099 | 77168857 | 1.39 |
| rs11896584 | 75494719 | 1.354 | rs4853299 | 77198160 | 1.383 |
| rs6737535 | 75508133 | 1.356 | rs1176784 | 77238189 | 1.321 |
| rs6546997 | 75512288 | 1.362 | rs2919058 | 77240716 | 1.312 |
| rs11126470 | 75521959 | 1.374 | rs6747704 | 77322231 | 1.31 |
| rs10201616 | 75529368 | 1.384 | rs1439721 | 77332404 | 1.308 |
| rs10208759 | 75534838 | 1.395 | rs10496205 | 77347215 | 1.297 |
| rs10496198 | 75534970 | 1.408 | rs6729161 | 77347478 | 1.298 |
| rs2588498 | 75562668 | 1.467 | rs446139 | 77391982 | 1.371 |
| rs730048 | 75668416 | 1.482 | rs17750913 | 77447635 | 1.425 |
| rs917235 | 75679327 | 1.482 | rs9309515 | 77471909 | 1.464 |
| rs6547014 | 75707296 | 1.481 | rs443237 | 77479953 | 1.474 |
| rs13426311 | 75709644 | 1.481 | rs424198 | 77492745 | 1.486 |
| rs1017051 | 75718785 | 1.481 | rs17014064 | 77494416 | 1.488 |
| rs10188239 | 75724524 | 1.495 | rs1922807 | 77516772 | 1.511 |
| rs7588016 | 75729848 | 1.485 | rs13013710 | 77520289 | 1.519 |
| rs3885981 | 75889777 | 1.447 | rs6749190 | 77523358 | 1.513 |
| rs13025724 | 76233412 | 1.155 | rs12990498 | 77529392 | 1.483 |
| rs1401838 | 76486648 | 1.136 | rs4561696 | 77535310 | 1.465 |
| rs1568378 | 76496775 | 1.184 | rs1882681 | 77537070 | 1.459 |
| rs1401841 | 76508069 | 1.229 | rs2861079 | 77546092 | 1.434 |
| rs11687001 | 76515912 | 1.224 | rs2861078 | 77551503 | 1.276 |
| rs7563300 | 76536768 | 1.186 | rs12463489 | 77636348 | 1.284 |
| rs963552 | 76650069 | 1.234 | rs4473409 | 77641404 | 1.285 |

| **SNP** | **BP** | **LOD** |
| --- | --- | --- |
| rs4485592 | 77711272 | 1.385 |
| rs10185229 | 77771863 | 1.491 |
| rs10520195 | 77799683 | 1.504 |
| rs7588183 | 77833614 | 1.521 |
| rs992870 | 77914582 | 1.606 |
| rs13398639 | 77949727 | 1.678 |
| rs13023861 | 78194015 | 1.35 |
| rs1358138 | 78350031 | 1.328 |
| rs10865433 | 78461609 | 1.302 |
| rs6725336 | 78493754 | 1.3 |
| rs13422559 | 78594435 | 1.292 |
| rs2102995 | 78625722 | 1.286 |
| rs2946582 | 78711378 | 1.272 |
| rs961331 | 78822897 | 1.218 |
| rs1521952 | 78866965 | 1.194 |
| rs1521964 | 78887425 | 1.19 |
| rs2177098 | 78914693 | 1.185 |
| rs13391901 | 78928609 | 1.183 |
| rs1521955 | 78957021 | 1.172 |
| rs17015944 | 79031775 | 1.144 |
| rs1261234 | 79036329 | 1.14 |
| rs11893407 | 79046697 | 1.12 |
| rs12614393 | 79095405 | 1.121 |
| rs283882 | 79196064 | 1.173 |
| rs2070708 | 79201790 | 1.186 |
| rs3819315 | 79218109 | 1.194 |
| rs892867 | 79218487 | 1.195 |
| rs986389 | 79259070 | 1.169 |
| rs17016644 | 79330069 | 1.184 |
| rs6710298 | 79332367 | 1.198 |
| rs2100290 | 79485855 | 1.231 |
| rs17017164 | 79540498 | 1.222 |
| rs1430642 | 79721176 | 1.126 |

**Chromosome 3, PRIM, peak LOD 2.50, support interval 15.06 Mb – 22.19 Mb**

| **SNP** | | **BP** | | **LOD** | | **SNP** | | **BP** | | **LOD** | |
| --- | --- | --- | --- | --- | --- | --- | --- | --- | --- | --- | --- |
| rs6442494 | | 15056356 | | 1.512 | | rs6778524 | | 16830957 | | 1.962 | |
| rs480644 | | 15099806 | | 1.532 | | rs4618210 | | 17099388 | | 2.05 | |
| rs17081778 | | 15148482 | | 1.555 | | rs4077616 | | 17148419 | | 2.06 | |
| rs1549983 | | 15169140 | | 1.555 | | rs4072191 | | 17153783 | | 2.061 | |
| rs17480477 | | 15236389 | | 1.555 | | rs4629317 | | 17155935 | | 2.061 | |
| rs6442511 | | 15315667 | | 1.556 | | rs6778609 | | 17163396 | | 2.115 | |
| rs826423 | | 15316605 | | 1.556 | | rs11916424 | | 17763993 | | 2.105 | |
| rs9837421 | | 15322297 | | 1.556 | | rs9861766 | | 18092469 | | 2.075 | |
| rs12637343 | | 15364820 | | 1.556 | | rs11713547 | | 18168657 | | 2.063 | |
| rs6796383 | | 15368074 | | 1.556 | | rs7653460 | | 18289870 | | 2.047 | |
| rs1563416 | | 15448768 | | 1.556 | | rs7433691 | | 18355448 | | 2.038 | |
| rs4395387 | | 15478059 | | 1.556 | | rs11716005 | | 18410506 | | 2.037 | |
| rs9880034 | | 15648273 | | 1.556 | | rs1359995 | | 18426691 | | 2.036 | |
| rs17041542 | | 15829047 | | 1.556 | | rs1328542 | | 18457562 | | 2.035 | |
| rs9815997 | | 16043358 | | 1.557 | | rs714141 | | 18470305 | | 2.035 | |
| rs2730362 | | 16063999 | | 1.557 | | rs6785093 | | 18578117 | | 2.031 | |
| rs6442577 | | 16077166 | | 1.557 | | rs13074328 | | 18581232 | | 2.031 | |
| rs17041846 | | 16077676 | | 1.557 | | rs336629 | | 18582175 | | 2.031 | |
| rs9861898 | | 16085957 | | 1.557 | | rs334960 | | 18585881 | | 2.029 | |
| rs12497859 | | 16089465 | | 1.557 | | rs7615587 | | 18791851 | | 2.021 | |
| rs4078148 | | 16138436 | | 1.557 | | rs6550679 | | 18820351 | | 2.018 | |
| rs7642249 | | 16158866 | | 1.557 | | rs11927855 | | 18860185 | | 2.018 | |
| rs9843983 | | 16159870 | | 1.557 | | rs4521250 | | 18877802 | | 2.017 | |
| rs11128786 | | 16208246 | | 1.557 | | rs4401379 | | 18905129 | | 2.06 | |
| rs6769977 | | 16216080 | | 1.767 | | rs11717739 | | 18906800 | | 2.062 | |
| rs11918238 | | 16216913 | | 1.76 | | rs9872957 | | 18925051 | | 2.073 | |
| rs3796301 | | 16217105 | | 1.758 | | rs13089058 | | 18932380 | | 2.094 | |
| rs905945 | | 16233654 | | 1.557 | | rs4973721 | | 19016093 | | 2.121 | |
| rs9848911 | | 16248562 | | 1.58 | | rs4101507 | | 19074098 | | 2.15 | |
| rs2063777 | | 16265171 | | 1.795 | | rs9873295 | | 19078743 | | 2.152 | |
| rs17042262 | | 16445201 | | 1.781 | | rs4101508 | | 19087138 | | 2.156 | |
| rs9817739 | | 16455831 | | 1.773 | | rs7612947 | | 19092122 | | 2.158 | |
| rs1517520 | | 16482329 | | 1.787 | | rs9847518 | | 19098536 | | 2.161 | |
| rs11128791 | | 16495439 | | 1.846 | | rs11926342 | | 19107555 | | 2.168 | |
| rs7623201 | | 16638687 | | 1.723 | | rs9855571 | | 19842571 | | 2.46 | |
| rs12490936 | | 16648449 | | 1.924 | | **rs9842115** | | **20378197** | | **2.497** | |
| rs4685368 | | 16657003 | | 1.924 | | rs594583 | | 20542787 | | 2.478 | |
| rs2162612 | | 16667079 | | 1.925 | | rs4858257 | | 20727496 | | 2.457 | |
| rs12492500 | | 16685565 | | 1.927 | | rs17810229 | | 20741564 | | 2.446 | |
| **SNP** | **BP** | | **LOD** | | **SNP** | | **BP** | | **LOD** | |  |
| rs1500425 | 20823052 | | 2.44 | | rs11129023 | | 21816908 | | 1.996 | |  |
| rs1391858 | 20903316 | | 2.426 | | rs2176081 | | 21836731 | | 1.991 | |  |
| rs6776153 | 20932855 | | 2.423 | | rs2670256 | | 21843055 | | 1.989 | |  |
| rs17007866 | 20959147 | | 2.419 | | rs17009773 | | 21860198 | | 1.915 | |  |
| rs13061016 | 20981298 | | 2.416 | | rs17009787 | | 21861188 | | 1.91 | |  |
| rs2030007 | 21018350 | | 2.405 | | rs2630807 | | 21867553 | | 1.875 | |  |
| rs1846072 | 21103194 | | 2.396 | | rs9310662 | | 21877981 | | 1.902 | |  |
| rs1370118 | 21165372 | | 2.375 | | rs12491696 | | 21889150 | | 1.973 | |  |
| rs7612811 | 21355529 | | 2.375 | | rs259432 | | 22032222 | | 2.141 | |  |
| rs341844 | 21442667 | | 2.374 | | rs9310678 | | 22033851 | | 2.142 | |  |
| rs17008793 | 21463749 | | 2.373 | | rs9310679 | | 22034288 | | 2.142 | |  |
| rs424842 | 21466943 | | 2.373 | | rs11914361 | | 22051141 | | 1.999 | |  |
| rs800621 | 21474217 | | 2.373 | | rs259460 | | 22061442 | | 2 | |  |
| rs163469 | 21480484 | | 2.373 | | rs1817442 | | 22079068 | | 1.951 | |  |
| rs163474 | 21499039 | | 2.373 | | rs1405827 | | 22094442 | | 1.849 | |  |
| rs9883288 | 21500142 | | 2.373 | | rs6793553 | | 22134900 | | 1.841 | |  |
| rs920118 | 21522353 | | 2.373 | | rs7618885 | | 22158442 | | 1.836 | |  |
| rs17009071 | 21597391 | | 2.373 | | rs7628166 | | 22161408 | | 1.835 | |  |
| rs9822059 | 21603134 | | 2.373 | | rs17010896 | | 22165906 | | 1.833 | |  |
| rs9822177 | 21624334 | | 2.226 | | rs7627634 | | 22166800 | | 1.831 | |  |
| rs17009299 | 21697704 | | 2.225 | | rs10513638 | | 22177157 | | 1.798 | |  |
| rs1080021 | 21726843 | | 2.077 | | rs11129048 | | 22178502 | | 1.794 | |  |
| rs2878599 | 21751128 | | 2.225 | | rs6786716 | | 22189527 | | 1.751 | |  |
| rs17009454 | 21765639 | | 2.002 | |  | |  | |  | |  |

**Chromosome 3, QTOT1, peak LOD 2.12, support interval 67.65 Mb – 76.08 Mb**

| **SNP** | **BP** | **LOD** | **SNP** | **BP** | **LOD** |
| --- | --- | --- | --- | --- | --- |
| rs4334654 | 67649944 | 1.235 | rs6777671 | 69331700 | 1.69 |
| rs17808417 | 67701187 | 1.225 | rs9840469 | 69357004 | 1.689 |
| rs4380422 | 67716304 | 1.224 | rs4530530 | 69362731 | 1.695 |
| rs17808754 | 67727171 | 1.224 | rs11713163 | 69378641 | 1.71 |
| rs13087619 | 67820390 | 1.218 | rs13322901 | 69383027 | 1.713 |
| rs1459914 | 67849940 | 1.206 | rs17005571 | 69390632 | 1.724 |
| rs6548756 | 67885556 | 1.187 | rs9813130 | 69426980 | 1.71 |
| rs9828872 | 67897427 | 1.18 | rs7635662 | 69504185 | 1.67 |
| rs4856894 | 67915983 | 1.182 | rs2872806 | 69506023 | 1.669 |
| rs4331688 | 67916439 | 1.19 | rs2200247 | 69512697 | 1.658 |
| rs1870541 | 67917315 | 1.19 | rs7643187 | 69534204 | 1.629 |
| rs6783207 | 67937025 | 1.194 | rs11916346 | 69536466 | 1.625 |
| rs9810776 | 67958497 | 1.205 | rs9859296 | 69598946 | 1.517 |
| rs7618552 | 68005036 | 1.205 | rs7652880 | 69615510 | 1.423 |
| rs9865881 | 68170195 | 1.204 | rs7653611 | 69683677 | 1.382 |
| rs6548962 | 68204481 | 1.204 | rs995829 | 69684170 | 1.382 |
| rs4311217 | 68346079 | 1.203 | rs7623679 | 69723492 | 1.368 |
| rs17047387 | 68367351 | 1.242 | rs4855317 | 69771141 | 1.345 |
| rs7632646 | 68379178 | 1.274 | rs9310167 | 69777387 | 1.344 |
| rs6764336 | 68389414 | 1.344 | rs12107193 | 69780146 | 1.343 |
| rs9826468 | 68403369 | 1.376 | rs9682528 | 69816900 | 1.336 |
| rs2313273 | 68405140 | 1.393 | rs17006368 | 69841898 | 1.334 |
| rs17231705 | 68415247 | 1.396 | rs12491686 | 69842880 | 1.333 |
| rs9837363 | 68418809 | 1.398 | rs7641148 | 69859414 | 1.339 |
| rs17047444 | 68419650 | 1.402 | rs1430604 | 69959009 | 1.341 |
| rs17047573 | 68493809 | 1.45 | rs9874957 | 69962856 | 1.341 |
| rs9865323 | 68499180 | 1.502 | rs7623610 | 70087971 | 1.34 |
| rs2175266 | 68622403 | 1.558 | rs7430257 | 70341367 | 1.495 |
| rs6766759 | 68660669 | 1.59 | rs17006864 | 70353609 | 1.525 |
| rs17247167 | 68811369 | 1.747 | rs7650154 | 70356673 | 1.533 |
| rs17048068 | 68985771 | 1.751 | rs6790613 | 70367934 | 1.515 |
| rs17048091 | 69008468 | 1.751 | rs1357268 | 70387228 | 1.444 |
| rs17048119 | 69023634 | 1.752 | rs6549324 | 70406905 | 1.482 |
| rs17048130 | 69034131 | 1.752 | rs9835020 | 70436014 | 1.491 |
| rs4315691 | 69060930 | 1.752 | rs805492 | 70488823 | 1.504 |
| rs7427984 | 69090935 | 1.751 | rs805486 | 70512999 | 1.503 |
| rs4855543 | 69120907 | 1.69 | rs13070827 | 70537224 | 1.556 |
| rs3853159 | 69220229 | 1.691 | rs9830720 | 70625786 | 1.827 |
| rs4473559 | 69305553 | 1.69 | rs6762974 | 70678539 | 1.967 |

| **SNP** | **BP** | **LOD** | **SNP** | **BP** | **LOD** |
| --- | --- | --- | --- | --- | --- |
| rs10511006 | 70779723 | 1.96 | rs9860628 | 72125690 | 1.329 |
| rs9825759 | 70851718 | 1.932 | rs4677071 | 72127451 | 1.33 |
| rs2573174 | 70965867 | 1.916 | rs11711611 | 72141635 | 1.349 |
| rs6791955 | 70967004 | 1.915 | rs9682418 | 72180217 | 1.372 |
| rs17008051 | 71081176 | 1.836 | rs6549436 | 72229210 | 1.488 |
| rs955109 | 71122084 | 1.833 | rs4677097 | 72264614 | 1.511 |
| rs13093086 | 71137852 | 1.828 | rs9865222 | 72292228 | 1.479 |
| rs17653411 | 71246868 | 1.833 | rs6784559 | 72304772 | 1.494 |
| rs10511014 | 71293800 | 1.809 | rs11707718 | 72307438 | 1.498 |
| rs4677029 | 71312833 | 1.766 | rs9829428 | 72310277 | 1.509 |
| rs1474272 | 71336883 | 1.765 | rs7628589 | 72314554 | 1.931 |
| rs17008343 | 71339788 | 1.764 | rs6776427 | 72538397 | 1.946 |
| rs6549383 | 71349575 | 1.761 | rs1438312 | 72560813 | 1.945 |
| rs880543 | 71361812 | 1.782 | rs2028240 | 72573442 | 1.944 |
| rs12374070 | 71434781 | 1.737 | rs2028241 | 72595592 | 1.925 |
| rs1653974 | 71434801 | 1.737 | rs923578 | 72703285 | 1.714 |
| rs1653973 | 71435130 | 1.737 | rs6810278 | 72721919 | 1.8 |
| rs13072512 | 71496735 | 1.736 | rs12632229 | 72722484 | 1.807 |
| rs6549387 | 71514829 | 1.735 | rs1873351 | 72733491 | 1.789 |
| rs10470554 | 71518064 | 1.735 | rs4677197 | 72750301 | 1.705 |
| rs6771130 | 71578698 | 1.734 | rs17010036 | 72761459 | 1.631 |
| rs9828619 | 71708813 | 1.734 | rs4677201 | 72765009 | 1.604 |
| rs830653 | 71730598 | 1.861 | rs1123121 | 72779850 | 1.495 |
| rs830617 | 71764640 | 1.652 | rs2322573 | 72792080 | 1.392 |
| rs7612407 | 71769998 | 1.583 | rs4677203 | 72796734 | 1.344 |
| rs9872923 | 71780451 | 1.504 | rs9985487 | 72829943 | 1.289 |
| rs11710870 | 71786809 | 1.447 | rs34031957 | 72882238 | 1.285 |
| rs704275 | 71791102 | 1.398 | rs3821561 | 72946194 | 1.28 |
| rs704285 | 71803256 | 1.249 | rs17043990 | 72963160 | 1.28 |
| rs3796226 | 71819797 | 1.252 | rs7611961 | 73014098 | 1.281 |
| rs17664166 | 71823126 | 1.26 | rs7621387 | 73017213 | 1.281 |
| rs6767160 | 71823758 | 1.262 | rs9883307 | 73073820 | 1.282 |
| rs7620998 | 71826496 | 1.269 | rs1532190 | 73086847 | 1.282 |
| rs1447904 | 71835097 | 1.36 | rs9310260 | 73185165 | 1.335 |
| rs2029013 | 71843212 | 1.359 | rs9850430 | 73320419 | 1.435 |
| rs1992872 | 71884402 | 1.295 | rs6775039 | 73324343 | 1.471 |
| rs6801209 | 72075453 | 1.329 | rs4676904 | 73332434 | 1.486 |
| rs12495849 | 72083709 | 1.331 | rs17751397 | 73343914 | 1.525 |
| rs1109569 | 72088125 | 1.339 | rs6763368 | 73370528 | 1.537 |

| **SNP** | **BP** | **LOD** | **SNP** | **BP** | **LOD** |
| --- | --- | --- | --- | --- | --- |
| rs1107251 | 73402675 | 1.548 | rs1526710 | 74188338 | 1.876 |
| rs504900 | 73403316 | 1.549 | rs9826787 | 74276806 | 1.977 |
| rs584012 | 73410050 | 1.553 | rs39693 | 74355365 | 2.049 |
| rs11916497 | 73413179 | 1.556 | rs862825 | 74418454 | 2.095 |
| rs6549523 | 73416725 | 1.576 | rs9867036 | 74427863 | 2.09 |
| rs3901920 | 73477301 | 1.561 | rs529655 | 74447501 | 2.102 |
| rs6777921 | 73508536 | 1.552 | **rs2044594** | **74474447** | **2.12** |
| rs7064 | 73514520 | 1.526 | rs549560 | 74481059 | 2.111 |
| rs6549540 | 73571360 | 1.538 | rs551595 | 74483819 | 2.108 |
| rs4677287 | 73598809 | 1.557 | rs11919225 | 74506415 | 2.08 |
| rs3855577 | 73610676 | 1.579 | rs6809915 | 74521771 | 2.045 |
| rs11128341 | 73618281 | 1.579 | rs7433810 | 74600103 | 2.011 |
| rs6783760 | 73619276 | 1.579 | rs13092046 | 74617642 | 2 |
| rs4676929 | 73641344 | 1.572 | rs13314386 | 74725367 | 1.949 |
| rs9310273 | 73698790 | 1.554 | rs4677425 | 74832125 | 1.915 |
| rs9879918 | 73735523 | 1.541 | rs1584333 | 74855022 | 1.776 |
| rs11926413 | 73738679 | 1.525 | rs12491189 | 75361048 | 1.894 |
| rs2063477 | 73843827 | 1.54 | rs11706176 | 75427740 | 1.91 |
| rs4676952 | 73867866 | 1.547 | rs9831266 | 75454761 | 1.918 |
| rs291545 | 73899427 | 1.563 | rs12490683 | 75461775 | 1.911 |
| rs291516 | 73927429 | 1.577 | rs6549723 | 75473191 | 1.803 |
| rs6796430 | 73950170 | 1.793 | rs6793001 | 75535790 | 1.651 |
| rs291504 | 73956783 | 1.832 | rs11712374 | 75540548 | 1.638 |
| rs291485 | 73961357 | 1.834 | rs35491846 | 75545672 | 1.639 |
| rs4676962 | 74011186 | 1.807 | rs12636221 | 75613585 | 1.601 |
| rs6780629 | 74015247 | 1.804 | rs11128446 | 75631852 | 1.588 |
| rs10460948 | 74028971 | 1.787 | rs7624950 | 75730577 | 1.487 |
| rs1405402 | 74053810 | 1.766 | rs2918531 | 75738560 | 1.477 |
| rs9824843 | 74075790 | 1.773 | rs11706032 | 75816778 | 1.403 |
| rs4416330 | 74094377 | 1.782 | rs13075591 | 76057642 | 1.275 |
| rs1405404 | 74131732 | 1.804 | rs7630881 | 76063871 | 1.271 |
| rs13065653 | 74141088 | 1.819 | rs9820706 | 76084636 | 1.213 |
| rs17710919 | 74148210 | 1.834 |  |  |  |

**Chromosome 5, QTOT2, peak LOD 2.76, support interval 122.43 Mb – 133.84 Mb**

| **SNP** | **BP** | **LOD** | **SNP** | **BP** | **LOD** |
| --- | --- | --- | --- | --- | --- |
| rs3924912 | 122434224 | 2.252 | rs930606 | 124216786 | 1.877 |
| rs11747907 | 122437333 | 2.256 | rs13362134 | 124277917 | 1.858 |
| rs929772 | 122448068 | 2.266 | rs17152193 | 124322347 | 1.852 |
| rs632678 | 122475673 | 2.326 | rs11950485 | 124414890 | 1.935 |
| rs17392426 | 122580725 | 2.373 | rs13162105 | 124511417 | 2.233 |
| rs17405383 | 122588069 | 2.379 | rs7725526 | 124513278 | 2.237 |
| rs17150263 | 122600015 | 2.389 | rs6868078 | 124517235 | 2.246 |
| rs35824 | 122626041 | 2.419 | rs930413 | 124521223 | 2.283 |
| rs1366337 | 122657165 | 2.433 | rs1439594 | 124547087 | 2.298 |
| rs675443 | 122672597 | 2.442 | rs9637914 | 124554493 | 2.303 |
| rs10519724 | 122740462 | 2.449 | rs1550370 | 124555972 | 2.311 |
| rs1382329 | 122967347 | 2.413 | rs7715337 | 124596199 | 2.33 |
| rs11748635 | 123232224 | 2.534 | rs1439607 | 124597370 | 2.33 |
| rs330676 | 123232291 | 2.523 | rs1439605 | 124601508 | 2.408 |
| rs12332303 | 123343240 | 2.452 | rs10036862 | 124693163 | 2.339 |
| rs2067077 | 123365743 | 2.413 | rs1517805 | 124835021 | 2.301 |
| rs17151189 | 123416329 | 2.429 | rs1026449 | 124856546 | 2.283 |
| rs7447381 | 123428973 | 2.435 | rs2125139 | 124891466 | 2.232 |
| rs2408118 | 123521585 | 2.431 | rs7702671 | 125002875 | 2.235 |
| rs160851 | 123615051 | 2.418 | rs6595621 | 125017478 | 2.238 |
| rs189781 | 123645746 | 2.404 | rs13360577 | 125018422 | 2.238 |
| rs10478612 | 123646236 | 2.403 | rs4836202 | 125035207 | 2.243 |
| rs11953978 | 123646807 | 2.402 | rs1352739 | 125052872 | 2.255 |
| rs2545699 | 123665310 | 2.209 | rs2029547 | 125071620 | 2.255 |
| rs895298 | 123762340 | 2.242 | rs10478672 | 125115338 | 2.231 |
| rs7705687 | 123774070 | 2.247 | rs17153316 | 125151926 | 2.184 |
| rs393547 | 123807199 | 2.149 | rs2431598 | 125155775 | 2.185 |
| rs4568357 | 123814751 | 2.084 | rs2161342 | 125157964 | 2.184 |
| rs4574539 | 123826877 | 1.988 | rs1991488 | 125189097 | 2.165 |
| rs6870395 | 123891033 | 1.914 | rs1175187 | 125527991 | 2.237 |
| rs1494671 | 123908750 | 1.866 | rs7722059 | 125546015 | 2.242 |
| rs6873640 | 123937399 | 1.786 | rs9327396 | 125603412 | 2.258 |
| rs1389881 | 123946447 | 1.842 | rs7718098 | 125649378 | 2.28 |
| rs11956421 | 123976629 | 1.839 | rs11740466 | 125761999 | 2.313 |
| rs12513661 | 124066150 | 1.861 | rs6897488 | 125797578 | 2.309 |
| rs4836112 | 124071620 | 1.879 | rs4426911 | 125806834 | 2.313 |
| rs6870431 | 124116778 | 1.883 | rs7736235 | 125816092 | 2.307 |
| rs10069971 | 124138545 | 1.886 | rs9327407 | 125825850 | 2.316 |
| rs6595552 | 124169985 | 1.887 | rs4836267 | 125852773 | 2.372 |

| **SNP** | **BP** | **LOD** | **SNP** | **BP** | **LOD** |
| --- | --- | --- | --- | --- | --- |
| rs2408700 | 125902424 | 2.297 | rs7735174 | 128440552 | 2.415 |
| rs12514417 | 125915614 | 2.27 | rs10520032 | 128529637 | 2.364 |
| rs4626335 | 125925310 | 2.269 | rs7356681 | 128534488 | 2.304 |
| rs4836277 | 125929340 | 2.272 | rs252671 | 128642950 | 2.41 |
| rs6870785 | 125933468 | 2.243 | rs7717089 | 128671135 | 2.454 |
| rs4836297 | 126057234 | 2.261 | rs1154826 | 128717895 | 2.474 |
| rs907195 | 126082966 | 2.267 | rs13184716 | 128720747 | 2.472 |
| rs2036846 | 126113611 | 2.273 | rs12658068 | 128723668 | 2.46 |
| rs9327420 | 126116148 | 2.276 | rs13174854 | 128766306 | 2.437 |
| rs12514001 | 126124760 | 2.316 | **rs6866597** | **128905516** | **2.757** |
| rs17165293 | 126211401 | 2.401 | rs10036775 | 129436699 | 2.392 |
| rs884241 | 126214856 | 2.47 | rs11741204 | 129721774 | 2.498 |
| rs10519925 | 126545605 | 2.471 | rs110411 | 129752270 | 2.508 |
| rs6892923 | 126574662 | 2.467 | rs12657641 | 130400822 | 2.527 |
| rs11957269 | 126646020 | 2.456 | rs17132283 | 131359558 | 2.566 |
| rs17165082 | 126664681 | 2.444 | rs3846726 | 131386898 | 2.59 |
| rs35524 | 126729521 | 2.436 | rs2074610 | 131757588 | 2.542 |
| rs11960775 | 126743105 | 2.389 | rs11242110 | 131771676 | 2.524 |
| rs6896658 | 126875997 | 2.25 | rs2706399 | 131895601 | 2.528 |
| rs9327443 | 126890601 | 2.22 | rs739719 | 131900764 | 2.524 |
| rs10491249 | 126909947 | 2.179 | rs2706364 | 131954697 | 2.48 |
| rs374499 | 126924959 | 2.147 | rs2522390 | 131958665 | 2.48 |
| rs10478774 | 126970672 | 2.077 | rs6884762 | 131966629 | 2.481 |
| rs13173672 | 127011681 | 2.112 | rs17691077 | 132071250 | 2.489 |
| rs791077 | 127014256 | 2.115 | rs2074529 | 132084046 | 2.494 |
| rs7711139 | 127019355 | 2.33 | rs7721926 | 132212331 | 2.492 |
| rs10519961 | 127169241 | 2.311 | rs42395 | 132242229 | 2.489 |
| rs245159 | 127276150 | 2.319 | rs4515334 | 132491340 | 2.514 |
| rs12188497 | 127390442 | 2.399 | rs7717377 | 132519461 | 2.502 |
| rs17608067 | 127604625 | 2.408 | rs2162769 | 132568008 | 2.477 |
| rs3805620 | 127634763 | 2.411 | rs959727 | 132594110 | 2.426 |
| rs2307108 | 127637901 | 2.414 | rs10069497 | 132640331 | 2.409 |
| rs153977 | 127705480 | 2.42 | rs7733176 | 132663834 | 2.416 |
| rs10073583 | 127962900 | 2.431 | rs10900816 | 132666892 | 2.42 |
| rs7715766 | 128030017 | 2.435 | rs6880320 | 132703652 | 2.414 |
| rs10072361 | 128045443 | 2.436 | rs4958121 | 132731599 | 2.403 |
| rs11951994 | 128046772 | 2.436 | rs25736 | 132763121 | 2.358 |
| rs11241972 | 128088048 | 2.439 | rs17685465 | 132767225 | 2.352 |
| rs6595861 | 128165956 | 2.449 | rs13164788 | 132767293 | 2.355 |

| **SNP** | **BP** | **LOD** |
| --- | --- | --- |
| rs7729507 | 132775664 | 2.389 |
| rs25872 | 132813476 | 2.394 |
| rs718410 | 132835571 | 2.384 |
| rs246961 | 132839877 | 2.382 |
| rs10463522 | 132842630 | 2.348 |
| rs26367 | 132917543 | 2.373 |
| rs13359225 | 133041002 | 2.554 |
| rs2867929 | 133133023 | 2.511 |
| rs258059 | 133138385 | 2.504 |
| rs10061439 | 133143838 | 2.505 |
| rs3909368 | 133161915 | 2.505 |
| rs245581 | 133188153 | 2.505 |
| rs1644293 | 133193833 | 2.505 |
| rs7378826 | 133249570 | 2.436 |
| rs10079371 | 133252173 | 2.427 |
| rs1618181 | 133268826 | 2.32 |
| rs6874189 | 133274100 | 2.279 |
| rs3892476 | 133277454 | 2.281 |
| rs7727689 | 133285364 | 2.095 |
| rs11956750 | 133395947 | 1.935 |
| rs244669 | 133444157 | 1.856 |
| rs30499 | 133469625 | 1.829 |
| rs2053026 | 133514357 | 1.827 |
| rs17165388 | 133665933 | 1.818 |
| rs328833 | 133810862 | 1.808 |
| rs1476095 | 133840609 | 1.8 |

**Chromosome 6, QTOT1, peak LOD 2.38, support interval 156.81 Mb – 159.48 Mb**

| **SNP** | **BP** | **LOD** | **SNP** | **BP** | **LOD** |
| --- | --- | --- | --- | --- | --- |
| rs2045138 | 156808166 | 1.695 | rs16900468 | 158181346 | 2.358 |
| rs9383810 | 156895326 | 1.708 | rs6914588 | 158222343 | 2.34 |
| rs6927999 | 156913513 | 1.71 | rs2490565 | 158246186 | 2.329 |
| rs288968 | 156916426 | 1.712 | rs11755474 | 158275006 | 2.317 |
| rs2767577 | 156938875 | 1.711 | rs577 | 158285023 | 2.314 |
| rs6916489 | 157016551 | 1.77 | rs9458975 | 158309729 | 2.307 |
| rs9384488 | 157051073 | 1.806 | rs9295289 | 158387494 | 2.358 |
| rs12208714 | 157065146 | 1.812 | rs11967187 | 158518568 | 2.363 |
| rs9397313 | 157083710 | 1.821 | rs628039 | 158537560 | 2.365 |
| rs11966916 | 157100441 | 1.849 | rs6909925 | 158554163 | 2.288 |
| rs287943 | 157265904 | 1.819 | rs7755193 | 158564847 | 2.219 |
| rs13216156 | 157432473 | 2.038 | rs262825 | 158598619 | 2.176 |
| rs10457101 | 157486606 | 2.097 | rs600752 | 158817440 | 2.12 |
| rs1007250 | 157503677 | 2.11 | rs17504391 | 158837152 | 2.106 |
| rs9397997 | 157508849 | 2.111 | rs7772700 | 158871063 | 2.066 |
| rs4256459 | 157705731 | 2.167 | rs16900884 | 158965710 | 2.061 |
| rs34773654 | 157758181 | 2.173 | rs2306749 | 159002261 | 2.059 |
| rs9457715 | 157876496 | 2.183 | rs317795 | 159004993 | 2.058 |
| rs17446605 | 157903128 | 2.182 | rs317802 | 159011319 | 2.057 |
| rs16900289 | 157926844 | 2.181 | rs9457437 | 159038966 | 2.046 |
| rs905924 | 157934243 | 2.182 | rs9347258 | 159153031 | 2.033 |
| rs16900291 | 157942683 | 2.184 | rs9457507 | 159236418 | 2.031 |
| rs672764 | 158010529 | 2.219 | rs9355255 | 159244960 | 2.03 |
| rs9355333 | 158012968 | 2.219 | rs12662184 | 159256206 | 2.011 |
| rs10945717 | 158017334 | 2.219 | rs7773373 | 159340135 | 1.791 |
| rs672786 | 158035775 | 2.22 | rs10945592 | 159407294 | 1.546 |
| rs9346896 | 158108536 | 2.345 | rs2485368 | 159446677 | 1.509 |
| rs16900421 | 158113232 | 2.349 | rs7772013 | 159448242 | 1.498 |
| rs1034058 | 158113713 | 2.297 | rs2451244 | 159470841 | 1.434 |
| **rs240642** | **158117314** | **2.377** | rs6905981 | 159480014 | 1.423 |

**Chromosome 19, QTOT2, peak LOD 2.15, support interval 59.42 Mb – 61.47 Mb**

| **SNP** | **BP** | **LOD** | **SNP** | **BP** | **LOD** |
| --- | --- | --- | --- | --- | --- |
| rs422948 | 59440823 | 1.428 | rs2278427 | 60109543 | 1.653 |
| rs2361797 | 59445355 | 1.53 | rs11880295 | 60124341 | 1.623 |
| rs3848606 | 59480568 | 1.542 | rs623383 | 60126387 | 1.618 |
| rs4806741 | 59502182 | 1.575 | rs8101856 | 60134286 | 1.662 |
| rs759818 | 59513631 | 1.615 | rs775879 | 60141567 | 1.872 |
| rs1205316 | 59531270 | 1.658 | rs7256285 | 60143396 | 1.884 |
| rs7259731 | 59533544 | 1.673 | rs12460693 | 60161024 | 2.034 |
| rs10419832 | 59541211 | 1.664 | rs3745905 | 60186444 | 2.088 |
| rs2241385 | 59541378 | 1.662 | **rs1671133** | **60198861** | **2.151** |
| rs2004431 | 59544422 | 1.501 | rs1654466 | 60270514 | 2.02 |
| rs10423634 | 59562724 | 1.507 | rs12460187 | 60278602 | 2.013 |
| rs10402121 | 59568721 | 1.508 | rs3786867 | 60376012 | 2.026 |
| rs1985840 | 59571888 | 1.515 | rs4806650 | 60417222 | 2.022 |
| rs11084332 | 59572623 | 1.515 | rs3826884 | 60429993 | 1.996 |
| rs7259643 | 59577504 | 1.524 | rs4806471 | 60451769 | 1.954 |
| rs12974194 | 59605946 | 1.58 | rs12611091 | 60492141 | 1.766 |
| rs1077829 | 59624545 | 1.607 | rs897799 | 60564003 | 1.716 |
| rs10418661 | 59629198 | 1.662 | rs6509940 | 60575691 | 1.705 |
| rs2008238 | 59684483 | 1.68 | rs3810168 | 60588521 | 1.705 |
| rs8111398 | 59694904 | 1.684 | rs11669732 | 60604144 | 1.7 |
| rs4806766 | 59705494 | 1.693 | rs1457109 | 60626996 | 1.711 |
| rs6509880 | 59709228 | 1.719 | rs35068131 | 60675666 | 1.738 |
| rs17606864 | 59744087 | 1.725 | rs512238 | 60724900 | 1.798 |
| rs12975662 | 59746666 | 1.725 | rs310451 | 60742656 | 1.833 |
| rs10420030 | 59756226 | 1.724 | rs2902925 | 60750549 | 1.847 |
| rs272422 | 59829848 | 1.707 | rs7250117 | 60757975 | 1.845 |
| rs400322 | 59864390 | 1.731 | rs693289 | 60802848 | 1.89 |
| rs1749313 | 59865354 | 1.733 | rs310465 | 60815558 | 1.888 |
| rs1654668 | 59866025 | 1.734 | rs188701 | 60868760 | 1.883 |
| rs731170 | 59868074 | 1.727 | rs2287831 | 60912256 | 1.882 |
| rs760186 | 59869741 | 1.693 | rs1020947 | 60922956 | 1.882 |
| rs11574599 | 59875226 | 1.639 | rs10417878 | 60941855 | 1.88 |
| rs1749320 | 59879091 | 1.64 | rs4801628 | 60984584 | 1.881 |
| rs1654660 | 59886999 | 1.641 | rs7249986 | 60993988 | 1.874 |
| rs6509899 | 59917647 | 1.644 | rs299175 | 61005340 | 1.875 |
| rs1325158 | 59918214 | 1.645 | rs2061748 | 61010606 | 1.874 |
| rs7256392 | 59919691 | 1.645 | rs7258704 | 61052475 | 1.857 |
| rs10409751 | 59941382 | 1.648 | rs436488 | 61056102 | 1.854 |
| rs649216 | 60016447 | 1.747 | rs9284431 | 61062570 | 1.693 |

| **SNP** | **BP** | **LOD** |
| --- | --- | --- |
| rs4801635 | 61064517 | 1.836 |
| rs302835 | 61105395 | 1.701 |
| rs434678 | 61138116 | 1.744 |
| rs7255090 | 61178385 | 1.756 |
| rs8102420 | 61188728 | 1.749 |
| rs306450 | 61192862 | 1.748 |
| rs1560691 | 61207146 | 1.744 |
| rs387220 | 61212834 | 1.775 |
| rs17608620 | 61214735 | 1.781 |
| rs2915961 | 61223437 | 1.761 |
| rs2574764 | 61226336 | 1.735 |
| rs306447 | 61241344 | 1.415 |
| rs729078 | 61270050 | 1.351 |
| rs8104997 | 61273712 | 1.338 |
| rs2087115 | 61275118 | 1.295 |
| rs516022 | 61301183 | 1.281 |
| rs672824 | 61328571 | 1.282 |
| rs659424 | 61329303 | 1.282 |
| rs8108902 | 61369903 | 1.298 |
| rs175530 | 61377275 | 1.302 |
| rs7258661 | 61383413 | 1.302 |
| rs10469312 | 61403013 | 1.286 |
| rs12709954 | 61408391 | 1.275 |
| rs10403183 | 61432998 | 1.249 |
| rs7254061 | 61454488 | 1.23 |
| rs8106321 | 61466373 | 1.195 |
